# Lys31/Lys90-dependent stabilization of GPX4 by Schisandrin lignans suppresses ferroptosis in drug-induced liver injury

**DOI:** 10.1038/s41420-026-03288-4

**Published:** 2026-08-17

**Authors:** Huan Lan, Yuting Zheng, Jiameng Li, Caihong Liu, Peng Wu, Lin An, Jinjun Wu, Caiyan Wang

**Affiliations:** State Key Laboratory of Traditional Chinese Medicine Syndrome, International Institute for Translational Chinese Medicine, Guangzhou University of Chinese Medicine, Guangzhou, Guangdong 510006 China

**Keywords:** Cell biology, Diseases

## Abstract

Drug-induced liver injury (DILI), particularly from isoniazid (INH), is a major clinical concern. Ferroptosis is implicated in DILI, yet direct GPX4 stabilizers with defined binding sites remain undiscovered. While *Schisandra chinensis* lignans exhibit hepatoprotective potential, their mechanistic interplay with ferroptosis remains unexplored. Here, we delineate a novel molecular axis by which Schisandrin A (SinA) and Schisandrin B (SinB) mitigate INH-induced hepatotoxicity through ferroptosis suppression. We demonstrate that SinA/SinB significantly attenuates hepatic injury markers (ALT/AST), iron overload, lipid peroxidation, and glutathione depletion in vitro and in vivo. Strikingly, GPX4 knockout abolished their protective effects, underscoring GPX4 as the pivotal target. ITC and SPR revealed high-affinity binding of SinA/SinB to GPX4, while molecular docking identified K31 and K90 as critical residues for GPX4 interaction. Mutagenesis studies confirmed that K31/K90 substitutions abolished SinA/SinB’s efficacy, highlighting a structure-dependent mechanism. Compound binding reduced the interaction between GPX4 and TRIM25, inhibited the ubiquitination of GPX4, as evidenced by Co-IP and MD. This stabilizes GPX4, suppresses lipid peroxidation/iron accumulation, and rescues INH-induced ferroptosis in vitro and in vivo. These findings establish a novel mechanism of GPX4 regulation and provide a structural blueprint for anti-ferroptotic drug design.

**Figure:**
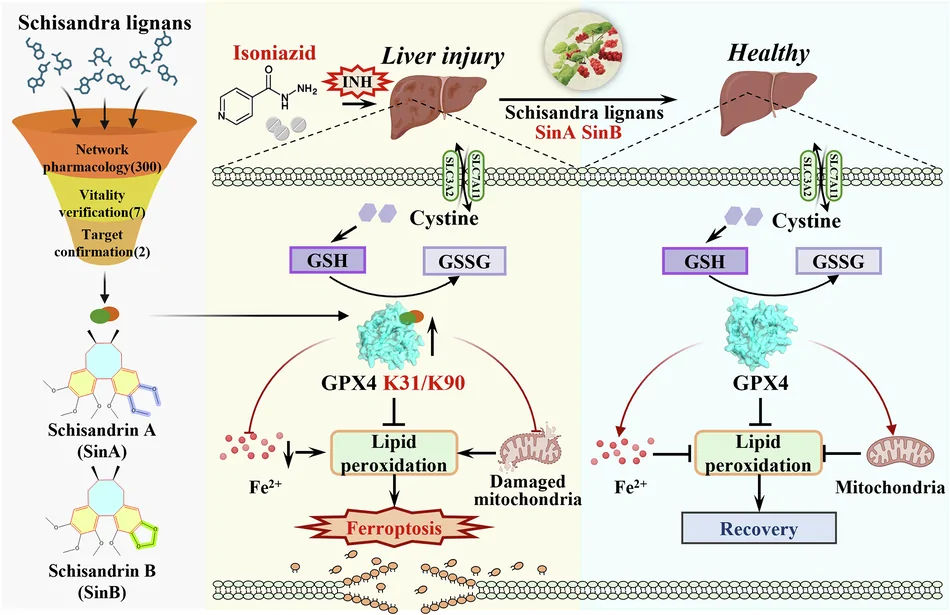

## Introduction

Drug-induced liver injury (DILI) refers to hepatobiliary damage caused by pharmaceuticals, herbal products, or dietary supplements [1]. With the global expansion and diversification of drug use, DILI has emerged as the leading cause of acute liver injury [2], which may progress to acute liver failure and even mortality in severe cases. As a first-line agent for tuberculosis treatment [3], INH-induced hepatotoxicity is one of the most common and clinically significant DILI events encountered during anti-tuberculosis therapy [4–7]. Its clinical manifestations range from asymptomatic liver enzyme elevation and hepatitis-like syndromes to acute liver failure and chronic liver injury, potentially leading to treatment interruption, severe hepatic damage, or even death [8–12]. Currently, agents such as bifendate, bicyclol, tiopronin, adenosylmethionine, and various vitamins are widely used to manage INH-induced liver injury [13–16]. However, these conventional hepatoprotective agents often provide incomplete protection and/or introduce additional side effects, including nausea, vomiting, and fatigue, and may even lead to more serious complications [17, 18]. Therefore, there is an urgent need to identify drugs with well-defined molecular targets that exhibit superior efficacy against DILI mechanisms while minimizing adverse effects, a critical issue in current clinical practice.

*Schisandra chinensis*, a well-established hepatoprotective reagent in clinical practice, contains Schisandrin lignans as its primary chemical components, which demonstrate diverse pharmacological activities [19–23]. Bifendate and Bicyclol, synthetic hepatoprotective agents derived from Schisandra lignans, are widely utilized clinically for their transaminase-lowering effects. Schisandrin A (SinA) and Schisandrin B (SinB) are two important schisandrin lignans with hepatoprotective activity. The primary structural distinction between SinA and SinB resides in the number and spatial arrangement of hydroxyl groups on the side chain. Specifically, SinA contains a single hydroxyl group at position C-8, whereas SinB possesses two hydroxyl groups located at positions C-8 and C-9, respectively. Research has demonstrated that SinA inhibits ferroptosis in cells and mitigates acetaminophen-induced acute liver injury by suppressing ROS accumulation through the Nrf2 and NF-κB signaling pathways. Additionally, it upregulates the expression of GPX4 and solute carrier family 7 member 11 (SLC7A11) while reducing the production of oxidized lipids [24, 25]. Pretreatment with SinB significantly improves bile acid metabolism and alleviates cholestatic liver injury [26]. The hepatoprotective mechanisms associated with this compound are closely linked to the upregulation of antioxidant enzyme expression, scavenging reactive oxygen species (ROS), inhibiting the release of inflammatory cytokines, reducing inflammatory cell infiltration, downregulating pro-apoptotic gene expression, and promoting hepatocyte regeneration, etc. [8, 27–30]. Notably, accumulating evidence suggests SinA/SinB target key pathways implicated in DILI, such as oxidative stress and inflammation. However, despite this broad hepatoprotective profile, the effectiveness of Schisandrin lignans, particularly SinA/SinB, in ameliorating INH-induced liver injury and its association with ferroptosis remains underexplored. Therefore, further investigation into its underlying molecular mechanisms in the context of INH-DILI is warranted.

Ferroptosis is a novel form of programmed cell death that differentiates itself from autophagy and apoptosis. This process is characterized by excessive iron-dependent lipid peroxidation, wherein the abnormal accumulation of lipid peroxides in the presence of Fe^2+^ leads to membrane disruption and subsequent irreversible cell death [31–34]. Glutathione peroxidase 4 (GPX4) serves as the primary enzymatic defense mechanism responsible for directly reducing and neutralizing toxic lipid peroxides into non-toxic lipid alcohols. This action effectively interrupts the oxidative chain reaction, thereby safeguarding cells against oxidative damage and ferroptosis [35, 36]. The activation of an amino acid antiporter, SLC7A11, favors intracellular GSH production to sustain GPX4 activity [37]. The most widely used ferroptosis inducers in preclinical research are inhibitors of GPX4, such as RSL-3, or SLC7A11, such as erastin [38, 39]. Significantly, ferroptosis has been increasingly recognized as a critical driver in the pathogenesis of DILI. Recent studies have found that DILI has pathological features related to ferroptosis. Biological processes such as redox imbalance, iron overload and lipid peroxidation associated with ferroptosis play an important role in the pathogenesis of DILI [40]. Some drugs or chemicals are metabolized in the liver into hepatotoxic substances, which deplete reduced GSH, impair the GSH-dependent reducing capacity of GPX4, lead to redox imbalance in hepatocytes, increase the levels of ROS and lipid peroxidation, and consequently cause hepatocyte damage; while other drugs promote ferritin autophagy, resulting in elevated iron levels and ferroptosis [40, 41]. However, the direct role of Schisandrin lignans, especially SinA/SinB, in modulating ferroptosis during INH-induced liver injury remains unexplored.

Consequently, this study conducts in vitro and in vivo experiments to investigate the mechanism of Schisandrin lignans, focusing on SinA/SinB, in inhibiting ferroptosis by stabilizing GPX4 and thereby specifically alleviating INH-induced liver injury. Findings of this research not only advance the development and application of Schisandrin lignans as targeted therapies for DILI but also provide experimental evidence for the formulation of novel DILI therapeutics aimed at ameliorating INH-induced liver injury through ferroptosis inhibition.

## Results

### SinA/SinB mitigate INH-induced hepatocyte injury

**Fig. 1:**
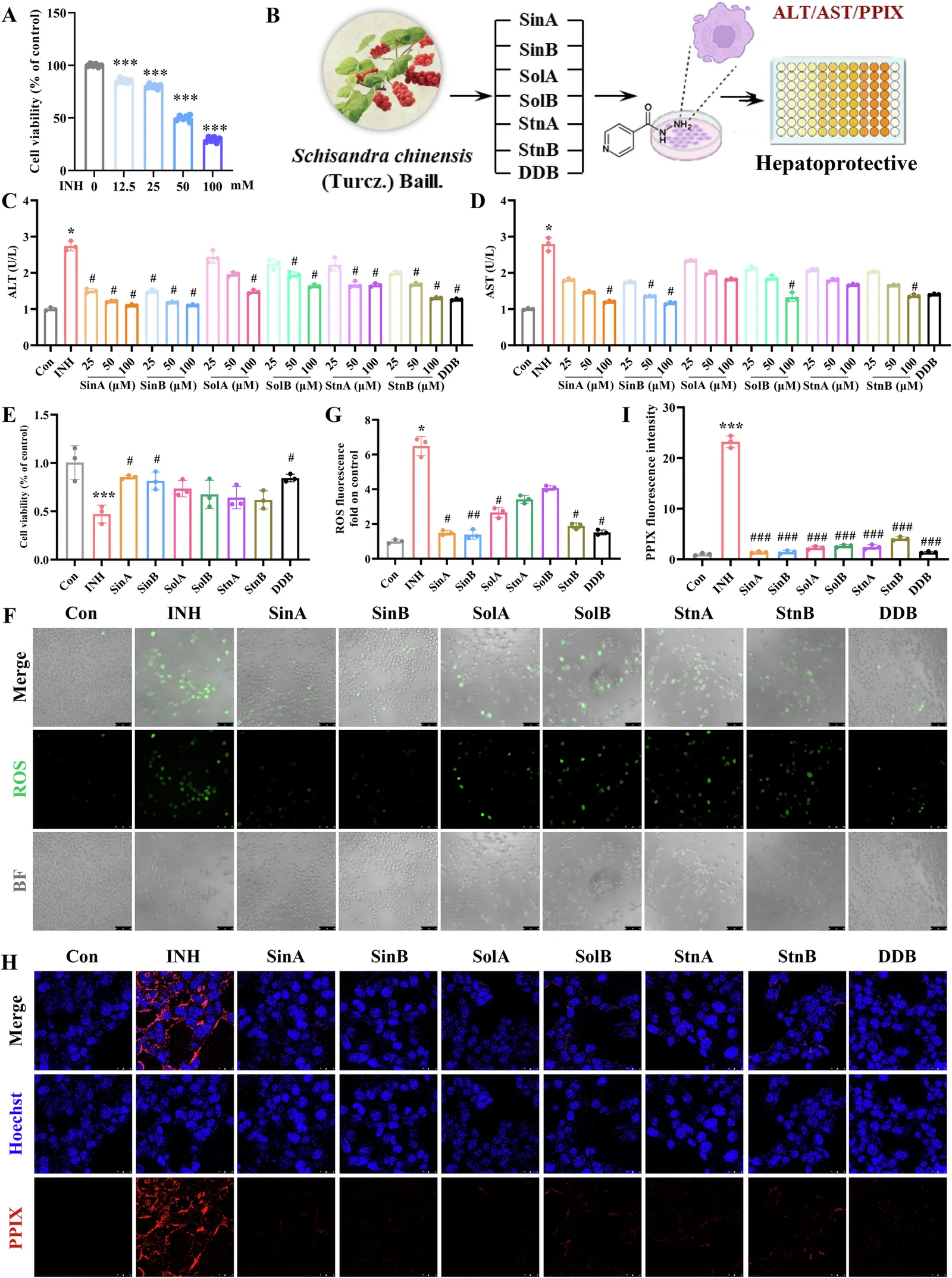
SinA/SinB protect against INH-induced hepatocyte injury in vitro. **A** Effects of INH on the cell viability of WRL68 cells as determined by MTT assay (*n* = 6). **B** Flowchart of drug screening in cells. **C**, **D** Evaluation of the degree of INH-induced liver injury improvement by SinA, SinB, SolA, SolB, StnA, StnB, and DDB in WRL68 cells using ALT and AST kits (*n* = 3). **E** Effects of SinA, SinB, SolA, SolB, StnA, StnB, and DDB on the cell viability of WRL68 cells as determined by MTT assay (*n* = 3). **F**, **G** Intracellular ROS levels in WRL68 cells were treated with SinA, SinB, SolA, SolB, StnA, StnB, and DDB after exposure to INH for 24 h. **H**, **I** Detection of the alleviation of INH-induced PPIX accumulation by SinA, SinB, SolA, SolB, StnA, StnB, and DDB in WRL68 cells using laser scanning confocal microscopy (scale bar: 25 μm, *n* = 3). Data were mean ± SD. *P < 0.05, **P < 0.01, ***P < 0.001 vs. control group, ^#^*P* < 0.05, ^###^*P* < 0.001 vs. INH group.

To establish a clinically relevant in vitro hepatotoxicity model, human hepatic WRL68 cells were exposed to increasing concentrations of INH (12.5–100 mM) for 24 h. MTT viability assays demonstrated a dose-dependent cytotoxic effect, with 50 mM INH inducing approximately 50% cell viability reduction (IC_50_ = 53.58 mM for WRL68; Fig. 1A). This concentration was subsequently selected as the optimal injury-inducing dose for mechanistic studies. To systematically evaluate the cytoprotective potential of Schisandra lignans, six structurally related compounds (SinA, SinB, SolA, SolB, StnA, and StnB) were administered at 25, 50, and 100 μM following INH challenge (Fig. 1B). Compared to the control, INH exposure significantly elevated the levels of ALT and AST in the culture supernatant. All tested lignans attenuated this enzyme leakage to varying degrees, with SinA/SinB exhibiting superior efficacy by restoring ALT and AST levels to near-baseline values (Fig. 1C, D). Consistent with this, MTT assays confirmed that SinA, SinB, and the positive control DDB (a synthetic hepatoprotective agent originally derived from Schisandra chinensis) significantly counteracted the INH-induced loss of cell viability (Fig. 1E). ROS detection revealed that SinA, SinB, SolA, StnB and DDB significantly reduced the INH-induced ROS elevation (Fig. 1F, G). Since INH inhibits ferrochelatase activity, leading to PPIX accumulation—a known contributor to cholestatic liver injury—we quantified intracellular PPIX. Laser confocal microscopy confirmed a significant increase in PPIX levels upon INH treatment. All six tested lignans and DDB significantly reduced PPIX content, with SinA/SinB showing the most potent effect (Fig. 1H, I). Based on these comprehensive findings, SinA/SinB were identified as the most effective hepatoprotective lignans for subsequent mechanistic investigation.

### SinA/SinB ameliorate INH-Induced liver injury via the ferroptosis pathway

**Fig. 2:**
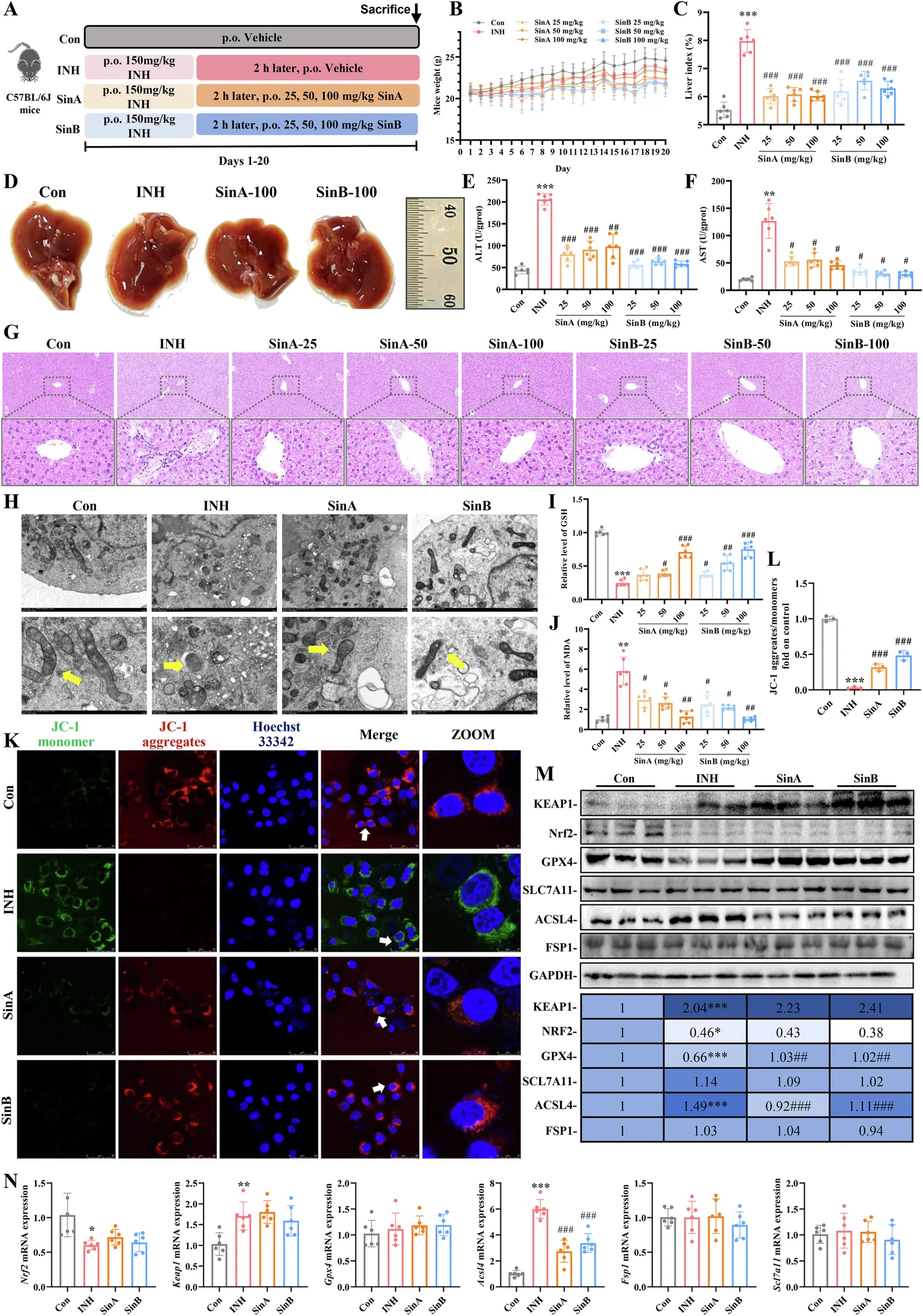
SinA/SinB could significantly inhibit ferroptosis. **A** Flowchart of animal drug administration. **B** Weight curve of mice (*n* = 6). **C** Liver index of mice (*n* = 6). **D** Images of mouse livers from various groups. **E** ALT levels in mouse liver tissue (*n* = 6). **F** AST levels in mouse liver tissue (*n* = 6). **G** H&E staining of mouse liver tissue (scale bar: 200 μm, *n* = 3). **H** Observation of mitochondrial morphology in WRL68 cells by TEM (upper scale bar: 2.0 μm, lower scale bar: 1.0 μm, *n* = 5). **I** GSH levels in mouse liver tissue (*n* = 6). **J** MDA Levels in mouse liver tissue (*n* = 6). **K**, **L** Detection of mitochondrial membrane potential levels in WRL68 cells using laser scanning confocal microscopy (scale bar: 50 μm, *n* = 3). **M** Western blot analysis of KEAP1, NRF2, GPX4, and SCL7A11 expression in mouse liver tissue. **N** RT-qPCR analysis of *Nrf2*, *Keap1*, *Gpx4, Acsl4, Fsp1*, and *Scl7a11* mRNA expression in mouse liver tissue. Data were mean ± SD. ^*^P < 0.05, ^**^P < 0.01, ^***^P < 0.001 vs. control group, ^#^*P* < 0.05, ^##^*P* < 0.01, ^###^*P* < 0.001 vs. INH group.

C57BL/6J mice were administered 150 mg/kg INH via oral gavage. Two hours after each INH dose, mice received SinA/SinB (25, 50, 100 mg/kg) for 20 consecutive days, while control and INH-only groups received no additional treatment (Fig. 2A). No significant differences in body weight were observed among groups during the experimental period (Fig. 2B). However, compared to the INH group, SinA/SinB-treated mice exhibited a marked reduction in liver index (Fig. 2C). Macroscopic examination showed that SinA/SinB intervention improved liver appearance, with high-dose groups displaying livers nearly comparable to the normal group (Fig. 2D). Serum biochemistry indicated that SinA/SinB treatment significantly lowered the INH-elevated levels of ALT and AST, confirming improved hepatic function (Fig. 2E, F). Histopathological analysis via H&E staining revealed that SinA/SinB treatment preserved near-normal liver lobule architecture, attenuated hepatocyte edema and hyaline degeneration, and minimized nuclear shrinkage compared to the INH group (Fig. 2G). To investigate the involvement of ferroptosis, a form of iron-dependent cell death, we examined its key hallmarks. TEM of WRL68 cells revealed that INH induced characteristic mitochondrial alterations of ferroptosis, including mitochondrial shrinkage, increased membrane density, and loss of cristae. These morphological damages were significantly ameliorated by treatment with SinA/SinB (Fig. 2H). Furthermore, SinA/SinB administration significantly suppressed lipid peroxidation, as evidenced by decreased MDA levels and elevated GSH content (Fig. 2I, J). Assessment of mitochondrial membrane potential using the JC-1 probe via laser confocal microscopy indicated that INH induced mitochondrial depolarization (increased JC-1 monomers), which was mitigated by SinA/SinB treatment (Fig. 2K, L). Western blot analysis of key ferroptosis-related proteins showed that SinA/SinB did not reverse the INH-induced increase in Keap1 or decrease in Nrf2 protein levels. And the protein level of FSP1 showed no significant difference among the various groups. However, SinA/SinB promoted the expression of GPX4 and inhibited the protein expression of ACSL4 (Fig. 2M). In addition, RT-PCR analysis of the mRNA levels of *Nrf2*, *Keap1*, *Gpx4*, *Fsp1*, and *Scl7a11* showed no significant differences before and after the administration of SinA/SinB treatments. SinA/SinB inhibited the mRNA level of *Acsl4* (Fig. 2N). These findings underscore the therapeutic potential of SinA/SinB in mitigating classical ferroptosis-driven liver injury.

### GPX4 is required for the anti-ferroptotic activity of SinA/SinB

**Fig. 3:**
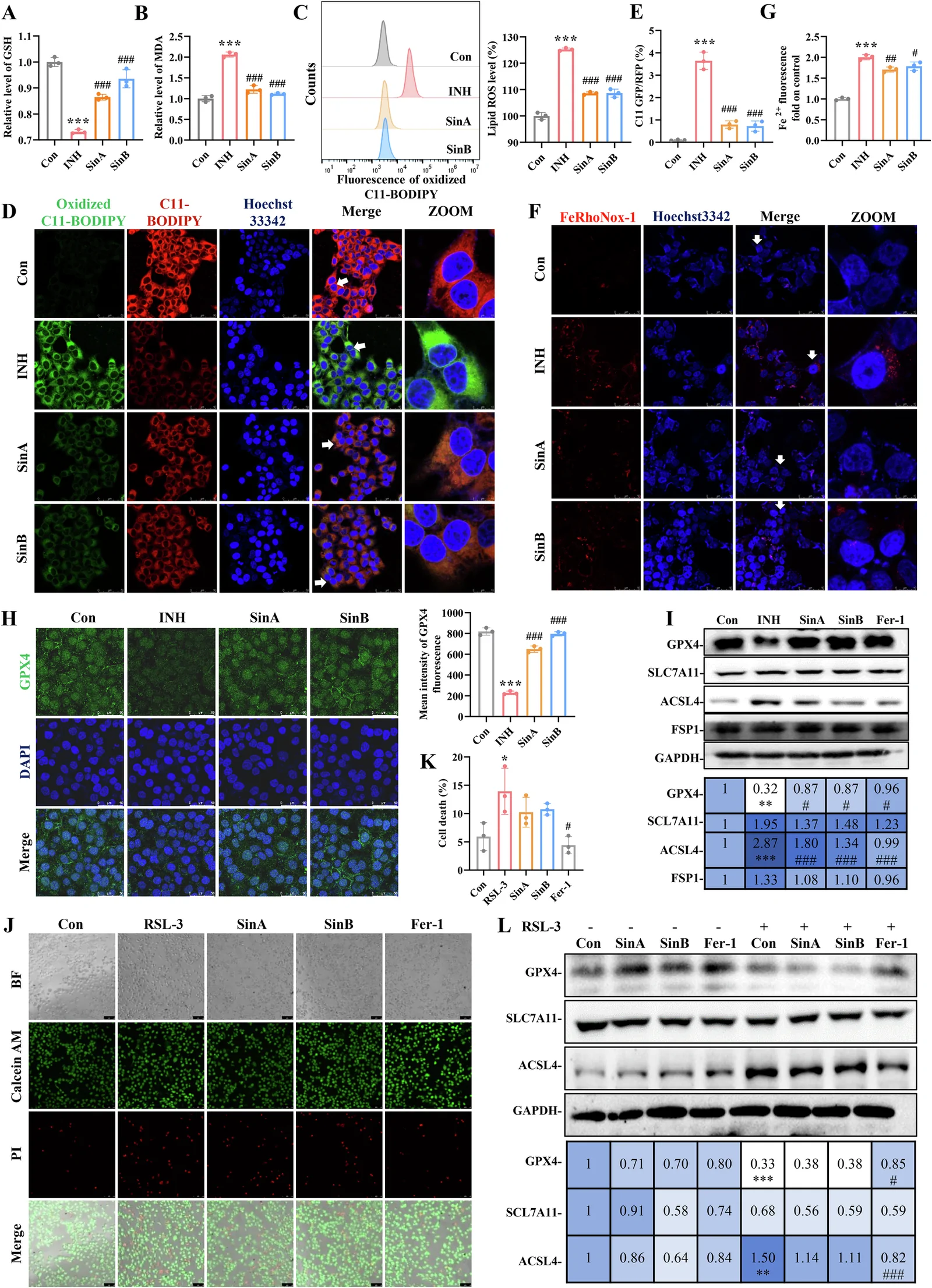
SinA/SinB inhibit ferroptosis in a GPX4-dependent manner. **A** Measurement of GSH levels in WRL68 cells (*n* = 3). **B** Measurement of MDA levels in WRL68 cells (*n* = 3). **C** Flow cytometry analysis of C11-BODIPY and oxidized C11-BODIPY fluorescence levels in WRL68 cells (*n* = 3). **D**, **E** Detection of C11-BODIPY and oxidized C11-BODIPY fluorescence levels in WRL68 cells using laser scanning confocal microscopy (scale bar: 50 μm, *n* = 3). **F**, **G** Detection of Fe²⁺ content in WRL68 cells using laser scanning confocal microscopy (scale bar: 50 μm, *n* = 3). **H** Laser scanning confocal microscopy for detection of GPX4 fluorescence intensity in the INH-induced WRL68 cells (scale bar: 50 μm, *n* = 3). **I** Western blot analysis of GPX4 protein expression levels in WRL68 cells. **J**, **K** Stained living and dead cells after incubation with RSL-3 (*n* = 3). **L** Western blot analysis of GPX4 protein expression levels in the RSL-3-induced WRL68 cells (*n* = 3). Data were mean ± SD. *P < 0.05, **P < 0.01, ^***^P < 0.001 vs. control group, ^##^*P* < 0.01, ^###^*P* < 0.001 vs. INH group.

Consistent with the in vivo data, INH depleted intracellular GSH and increased MDA levels in WRL68 cells and AML12 cells, and these effects were reversed by SinA/SinB (Fig. 3A, B and Fig. S1A, B). Given the interplay between mitochondrial dysfunction and lipid peroxidation in ferroptosis, we directly assessed lipid peroxidation dynamics. Flow cytometry analysis further confirmed that SinA/SinB reduced INH-induced elevation of lipid ROS (Fig. 3C). Confocal microscopy confirmed increased lipid peroxidation in INH-treated WRL68 cells and AML12 cells, which was significantly reduced by 50 μM SinA/SinB treatment (Fig. 3D, E and Fig. S1C, D). Ferroptosis execution critically depends on the accumulation of labile iron. Using the Fe²⁺-sensitive probe FeRhoNox-1, we observed a significant increase in intracellular Fe²⁺ upon INH treatment, which was effectively suppressed by SinA/SinB (Fig. 3F, G). Immunofluorescence analyses demonstrated that INH downregulated GPX4 protein expression, an effect that was reversed by SinA/SinB treatment (Fig. 3H). Western blot analyses demonstrated that INH decreased GPX4 protein expression and increased ACSL4 protein expression. This effect could be reversed by SinA/SinB treatment in WRL68 cells and AML12 cells. Western blot showed that the protein levels of FSP1 and SCL7A11 showed no significant difference among the various groups in WRL68 cells and AML12 cells (Fig. 3I and Fig. S1E). To ascertain the dependence on GPX4, we employed specific ferroptosis inducers. Ferroptosis inducer Erastin increased cell death, and this effect was reversed by SinA, SinB, or ferroptosis inhibitor Fer-1 (Fig. S2A). In contrast, RSL-3, a direct GPX4 inhibitor, also induced cell death, but its effect was not rescued by SinA/SinB (Fig. 3J, K). Similarly, Western blot showed that SinA/SinB could reverse the Erastin-induced decrease in GPX4 protein but not the decrease caused by RSL-3 (Fig. S2B and Fig. 3L). These findings indicate that SinA/SinB inhibit ferroptosis in a manner dependent on GPX4 function.

### SinA/SinB fail to exert hepatoprotective effects after GPX4 knockdown in vitro

**Fig. 4:**
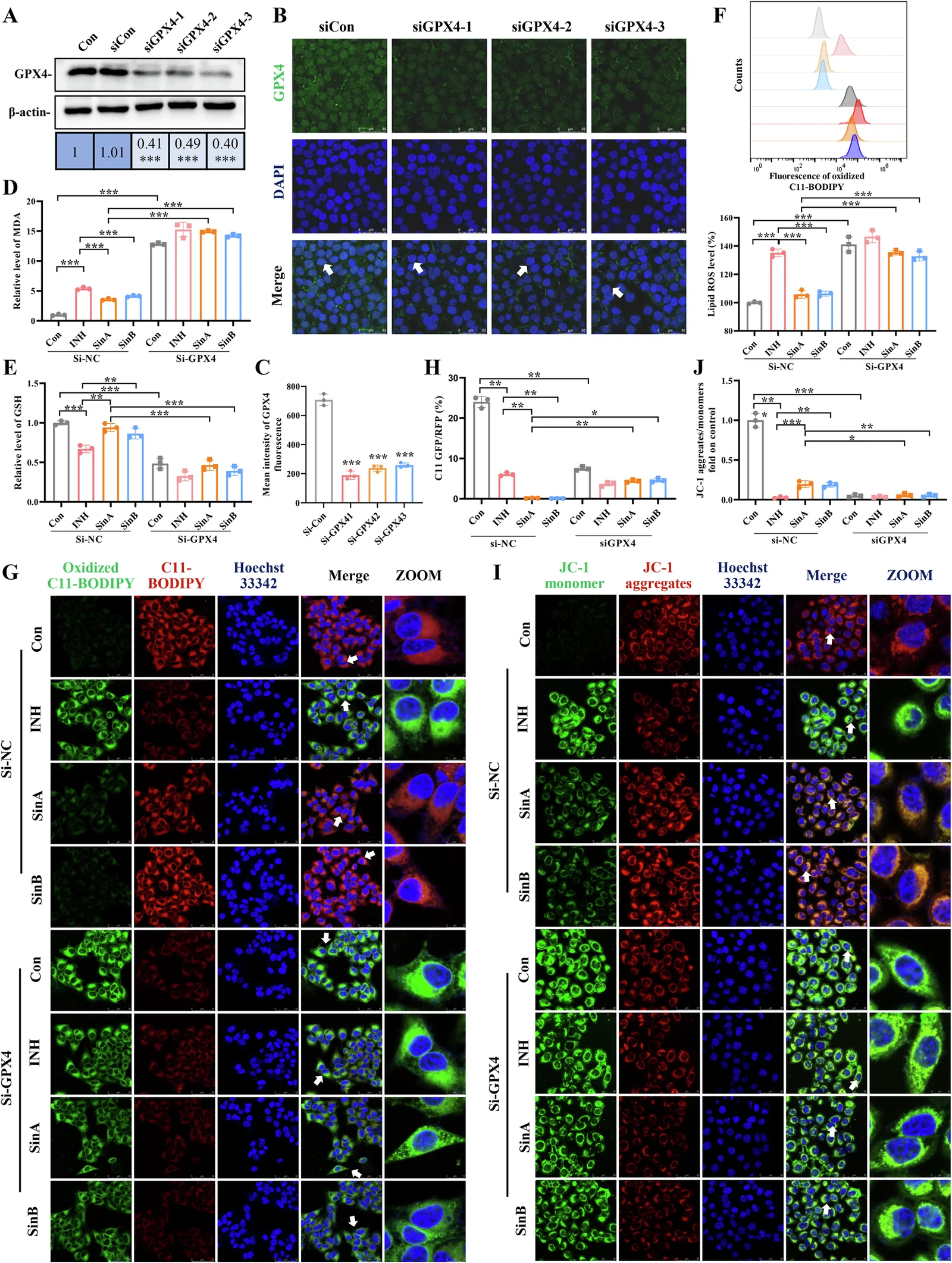
GPX4 knockdown abolishes the hepatoprotective effects of SinA/SinB. **A** Western blot assessment of GPX4 knockdown efficiency in WRL68 cells. **B**, **C** Laser scanning confocal microscopy for verification of GPX4 knockdown efficiency in WRL68 cells (scale bar: 50 μm, *n* = 3). **D** Measurement of MDA levels in WRL68 cells after GPX4 knockdown (*n* = 3). **E** Measurement of GSH levels in WRL68 cells after GPX4 knockdown (*n* = 3). **F** Flow cytometry analysis of C11-BODIPY and oxidized C11-BODIPY fluorescence levels in WRL68 cells after GPX4 knockdown (*n* = 3). **G**, **H** Detection of C11-BODIPY and oxidized C11-BODIPY fluorescence levels in WRL68 cells after GPX4 Knockdown using laser scanning confocal microscopy (scale bar: 50 μm, *n* = 3). **I**, **J** Detection of mitochondrial membrane potential levels in WRL68 cells after GPX4 Knockdown using laser scanning confocal microscopy (scale bar: 50 μm, *n* = 3). Data were mean ± SD. ^*^P < 0.05, ^**^P < 0.01, ^***^P < 0.001 vs. corresponding groups.

To establish the essential role of GPX4 in mediating the protective effects of SinA/SinB, we generated GPX4-knockdown (KD) WRL68 cells using siRNA. Western Blot and immunofluorescence results showed that GPX4 protein expression was significantly reduced after transfection with siGPX4, indicating successful knockdown (KO efficiency >85%, *p* < 0.001; Fig. 4A–C). SinA/SinB could alleviate the levels of MDA in wild-type (WT) cells caused by INH, but administration of SinA/SinB after GPX4 knockout failed to lower the MDA levels (Fig. 4D). Consistently, treatment with SinA/SinB significantly increased the GSH levels reduced by INH, but administration of SinA/SinB after GPX4 knockout failed to reverse the GSH levels (Fig. 4E). Through flow cytometry detection, it was found that treatment with SinA/SinB significantly decreased the lipid ROS levels increased by the INH group. However, the lipid ROS levels failed to reverse administration of SinA/SinB after GPX4 knockout (Fig. 4F). To assess lipid peroxidation, we used the C11-BODIPY probe and observed that in GPX4-deficient cells, SinA/SinB cannot inhibit the increased lipid peroxidation reduced by INH (Fig. 4G, H). JC-1 staining results showed that GPX4 knockout eliminated the protective effect of SinA/SinB on mitochondrial integrity (Fig. 4I, J). GPX4 ablation completely abolished the hepatoprotective effects of SinA/SinB. These results demonstrate that GPX4 is indispensable for the anti-ferroptotic activity of SinA/SinB.

### GPX4 upregulation mediates SinA/SinB-dependent ferroptosis resistance

**Fig. 5:**
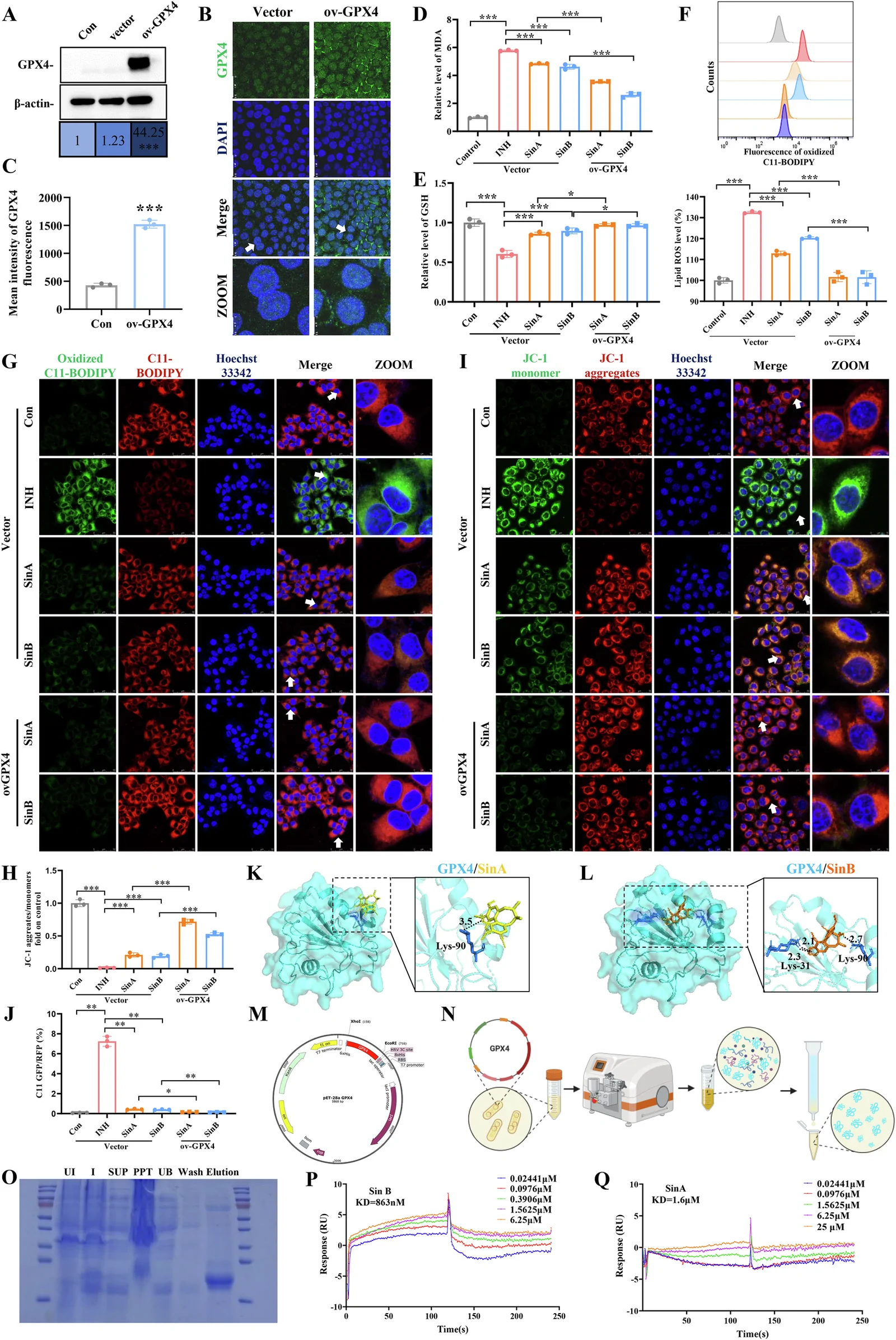
GPX4 overexpression enhances the anti-ferroptotic effects of SinA/SinB. **A** Western blot assessment of GPX4 overexpression efficiency in WRL68 cells. **B**, **C** Laser scanning confocal microscopy for verification of GPX4 overexpression efficiency in WRL68 cells (scale bar: 50 μm, *n* = 3). **D** Measurement of GSH levels in WRL68 cells after GPX4 overexpression (*n* = 3). **E** Measurement of MDA levels in WRL68 cells after GPX4 overexpression (*n* = 3). **F** Flow cytometry analysis of C11-BODIPY and oxidized C11-BODIPY fluorescence levels in WRL68 cells after GPX4 overexpression (*n* = 3). **G**, **H** Laser scanning confocal microscopy detection of C11-BODIPY and oxidized C11-BODIPY fluorescence levels in WRL68 cells after GPX4 overexpression (scale bar: 50 μm, *n* = 3). **I**, **J** Laser scanning confocal microscopy detection of mitochondrial membrane potential levels in WRL68 cells after GPX4 overexpression (scale bar: 50 μm, *n* = 3). **K** Predicted binding models of SinA within the GPX4 protein structure from molecular docking simulations. Key interacting residues are labeled. **L** Predicted binding models of SinB within the GPX4 protein structure from molecular docking simulations. Key interacting residues are labeled. **M** Diagram of the pET28a plasmid with the Gpx4 gene. The Gpx4 gene is marked in red. **N** Flowchart of protein extraction process. **O** Graph of GPX4 protein expression. **P** The affinities of GPX4 binding with SinA were detected by Biacore. **Q** The affinities of GPX4 binding with SinB were detected by Biacore. Data were mean ± SD. ^*^P < 0.05, ^**^P < 0.01, ^***^P < 0.001 vs. corresponding groups.

We overexpressed GPX4 in WRL68 cells. Western blot and immunofluorescence results showed that GPX4 protein expression was significantly increased (Fig. 5A–C). In GPX4-overexpressing cells, SinA/SinB treatment led to a more pronounced reduction in INH-induced MDA levels (Fig. 5D) and a more effective restoration of GSH (Fig. 5E) compared to WT cells. GPX4-OE also enhanced the ability of SinA/SinB to lower lipid ROS (Fig. 5F), reduce lipid peroxidation (C11-BODIPY assay; Fig. 5G, H), and preserve mitochondrial membrane potential (JC-1 assay; Fig. 5I, J). Molecular docking work to identify the preferred binding site and theoretical binding mode of Schisandra lignans to GPX4 using the Autodock1.1.2 software. All the ligands showed similar binding energies, ranging from −5.3 to −6.8 kcal/mol, but they bind to different sites of the protein. Particularly, SinA shows abundant interactions with GPX4, centering at Lys90, whereas SinB makes contacts with Lys31 and Lys90 (Fig. 5K, L). We constructed a GPX4 expression plasmid for subcloning into the pET28α vector to overexpress the GPX4 protein using an *E. coli* expression system (Fig. 5M). The extraction process and expression situation of the protein GPX4 are shown in Fig. 5N, O. SPR indicated that the affinities of SinA/SinB were 1.6 and 863 nM, respectively (Fig. 5P, Q). Indeed, these findings underscore GPX4 as a central mediator of SinA/SinB in counteracting ferroptosis and alleviating INH-induced hepatic injury by modulating oxidative stress and mitochondrial dysfunction.

### SinA/SinB stabilizes GPX4 by inhibiting its ubiquitination-proteasome degradation

**Fig. 6:**
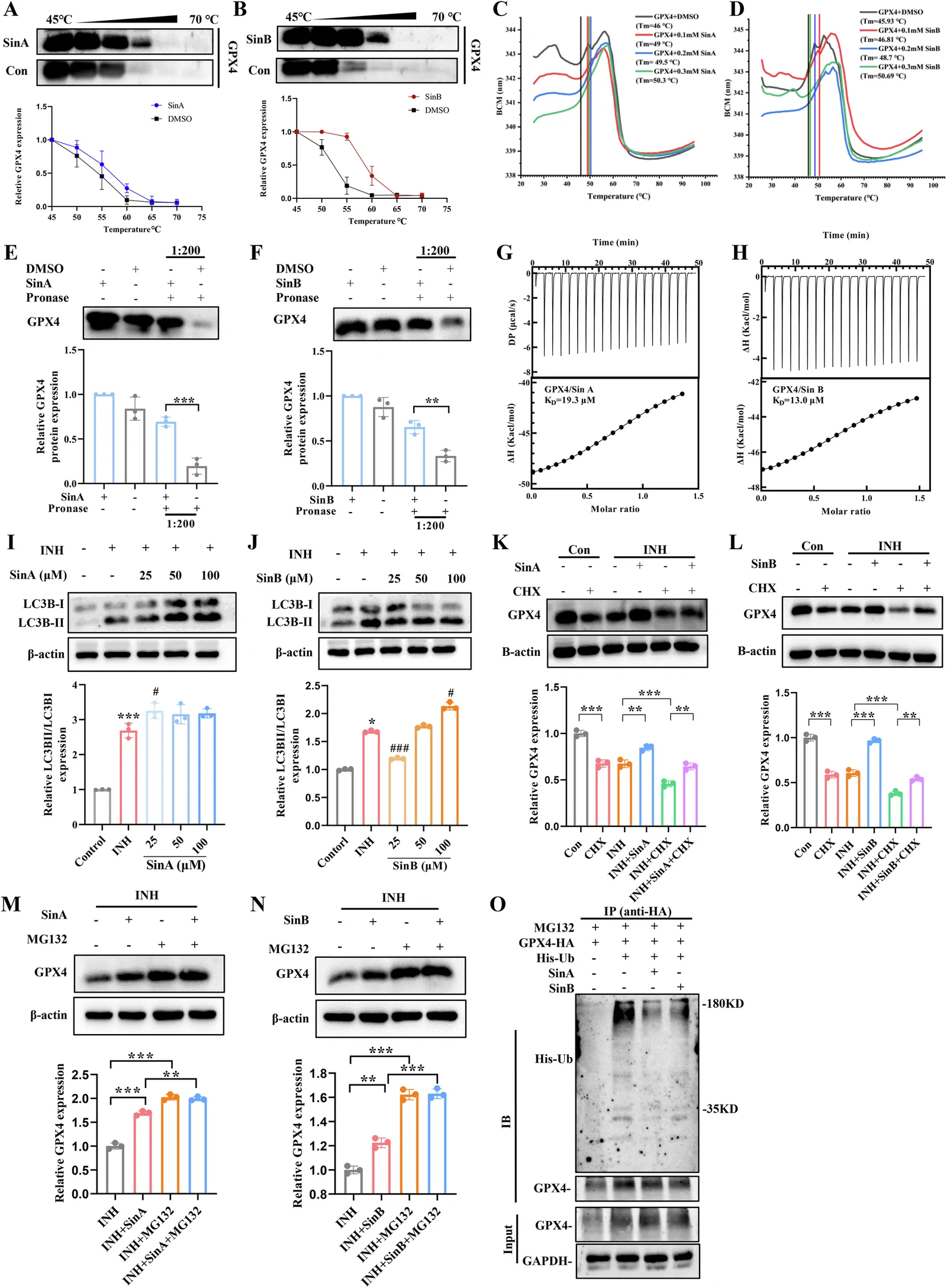
SinA/SinB stabilize GPX4 protein by inhibiting its ubiquitin-mediated degradation. **A** CETSA experiments of SinA with GPX4 protein. **B** CETSA experiments of SinB with GPX4 protein. **C**, **D** Thermal stabilities of GPX4 with SinA/SinB by DSF assay. GPX4 represents the group that GPX4 protein without the peptide, and is colored in black. The GPX4 protein incubated with is colored in Black, red and blue, respectively. The ΔTm values are summarized in the table and represent the Tm values of the peptide-added group minus those of the GPX4 group (*n* = 3). **E** DARTs experiments of SinA with GPX4 protein. **F** DARTs experiments of SinB with GPX4 protein. **G**, **H** Investigation of the binding affinity of different ligands and GPX4. Representative ITC titration profiles of GPX4 with SinA/SinB. The top graphs represent the raw ITC thermograms, and the bottom graphs represent the fitted binding isotherms. The corresponding *K*_d_ value has been marked in the figure. ITC experiments of SinA/SinB with GPX4. **I**, **J** Western blot analysis of LC3BII/LC3BI expression in WRL68 cells (*n* = 3). **K**, **L** Western blot analysis of the effects of SinA/SinB on GPX4 expression levels in WRL68 cells in the presence of CHX. **M**, **N** Western blot analysis of the effects of SinA/SinB on GPX4 expression levels in WRL68 cells in the presence of MG132. **O** Western blot analysis of GPX4 ubiquitination levels in WRL68 cells under the influence of SinA/SinB. Data were mean ± SD. ^**^P < 0.01, ^***^P < 0.001^, ##^*P* < 0.01 vs. corresponding groups. NS not significant.

To assess whether changes in GPX4 protein stability caused the decrease in GPX4 expression, the CETSA indicated that SinA/SinB increased the thermal stability of GPX4 (Fig. 6A, B and Fig. S1F, G). We then analyzed the stability of GPX4 with SinA/SinB. The melting temperature (Tm) of GPX4 with SinA/SinB was 51.59 and 52 °C compare to 46 °C for GPX4 with DMSO (Fig. 6C, D). Correspondingly, DARTS showed that the digestion of GPX4 by protease E was attenuated in the presence of SinA/SinB (Fig. 6E, F). ITC assay further showed that the binding *K*d values of SinA/SinB to GPX4 were 1.19 and 9.09 μM, respectively (Fig. 6G, H). Western blotting ruled out the involvement of mitophagy, as indicated by unaltered LC3BII/LC3BI ratios (Fig. 6I, J). Co-treatment with the protein synthesis inhibitor cycloheximide (CHX) did not prevent SinA/SinB-induced GPX4 accumulation (Fig. 6K, L), indicating a post-translational mechanism. Co-treatment with the proteasome inhibitor MG132 synergistically increased GPX4 protein levels with SinA/SinB (Fig. 6M, N), implicating the ubiquitin-proteasome system. Consistently, Co-IP assays demonstrated that SinA/SinB significantly reduced ubiquitination of GPX4 (Fig. 6O). Collectively, these data indicate that SinA/SinB stabilize GPX4 protein by inhibiting its ubiquitin-mediated proteasomal degradation.

### K31 and K90 are essential for SinA/SinB binding and GPX4 stabilization

**Fig. 7:**
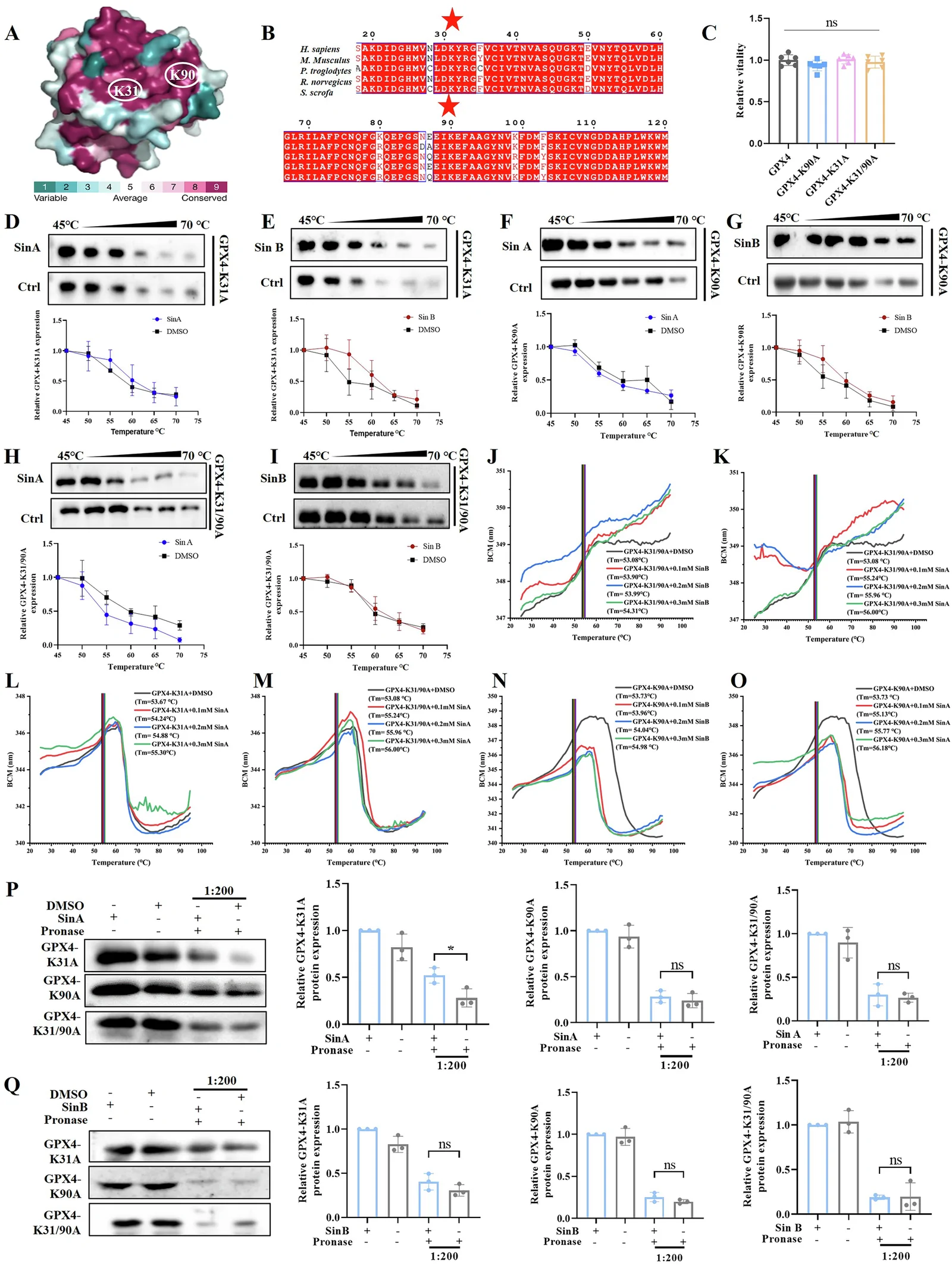
K31 and K90 residues of GPX4 are critical for binding and stabilization by SinA/SinB. **A** Conservative analysis of GPX4. **B** Multiple sequence alignment of GPX4 from different species. *H. sapiens*, *M. musculus*, *P. troglodytes, R. norvegicus*, *and S. scrofa represent Homo sapiens*, *Mus musculus*, *Pan troglodytes*, *Rattus norvegicus*, and *Sus scrofa*, respectively. The red background represents extremely conserved residues, and the red font represents relatively conserved residues. **C** Determination of GPX4 enzyme activity. **D**–**I** CETSA experiments of SinA/SinB with GPX4-K31A, GPX4-K90A and GPX4-K31A/K90A. **J**–**O** Thermal stabilities of GPX4-K31A, GPX4-K90A, and GPX4-K31A/K90A with two different ligands by DSF assay. The ΔTm values are summarized in the table and represent the Tm values of the peptide-added group minus those of the GPX4-K31A, GPX4-K90A, and GPX4-K31A/K90A group (*n* = *3*). **P**, **Q** DARTs experiments of SinA/SinB with GPX4-K31A, GPX4-K90A, and GPX4-K31A/K90A proteins. Data were mean ± SD. ^*^P < 0.05 vs. corresponding groups. ns not significant.

Furthermore, the ConSurf Server (https://consurf.tau.ac.il) was used for sequence conservation analysis of GPX4 (Fig. 7A). A multiple sequence alignment of GPX4 from different species was conducted. *H. sapiens, M. musculus, P. troglodytes, R. norvegicus*, and *S. scrofa* respectively stand for *Homo sapiens, Mus musculus, Pan troglodytes, Rattus norvegicus*, and *Sus scrofa*. The red background represents extremely conserved residues, and the red font represents relatively conserved residues. Meanwhile, by comparing GPX4 sequences from different species, we found that conserved Lys31 and Lys90 could play a key role (Fig. 7B). To test their functional importance, we generated GPX4 mutants: K31A, K90A, and the double mutant K31A/K90A. The enzymatic activity of GPX4 itself is retained after the K31/K90 mutations (Fig. 7C). CETSA experiments revealed that SinA increased the thermal stability of GPX4 WT and the K31A mutant, but not the K90A or double mutant (Fig. 7D, F, H). In contrast, SinB failed to stabilize any of the single or double mutants (Fig. 7E, G, I). No significant difference in Tm was observed between GPX4 mutant bound to SinA/SinB and that bound to DMSO (Fig. 7J–O). DARTS assays corroborated these findings: SinA protected WT and K31A GPX4 from proteolysis but not the K90A or double mutant (Fig. 7P). SinB did not significantly protect any of the mutants from digestion (Fig. 7Q). These results identify Lys90 as a critical site for SinA binding and stabilization, while both Lys31 and Lys90 appear important for SinB’s interaction with GPX4.

### Mutations at K31 and K90 attenuate the anti-ferroptotic efficacy of SinA/SinB

**Fig. 8:**
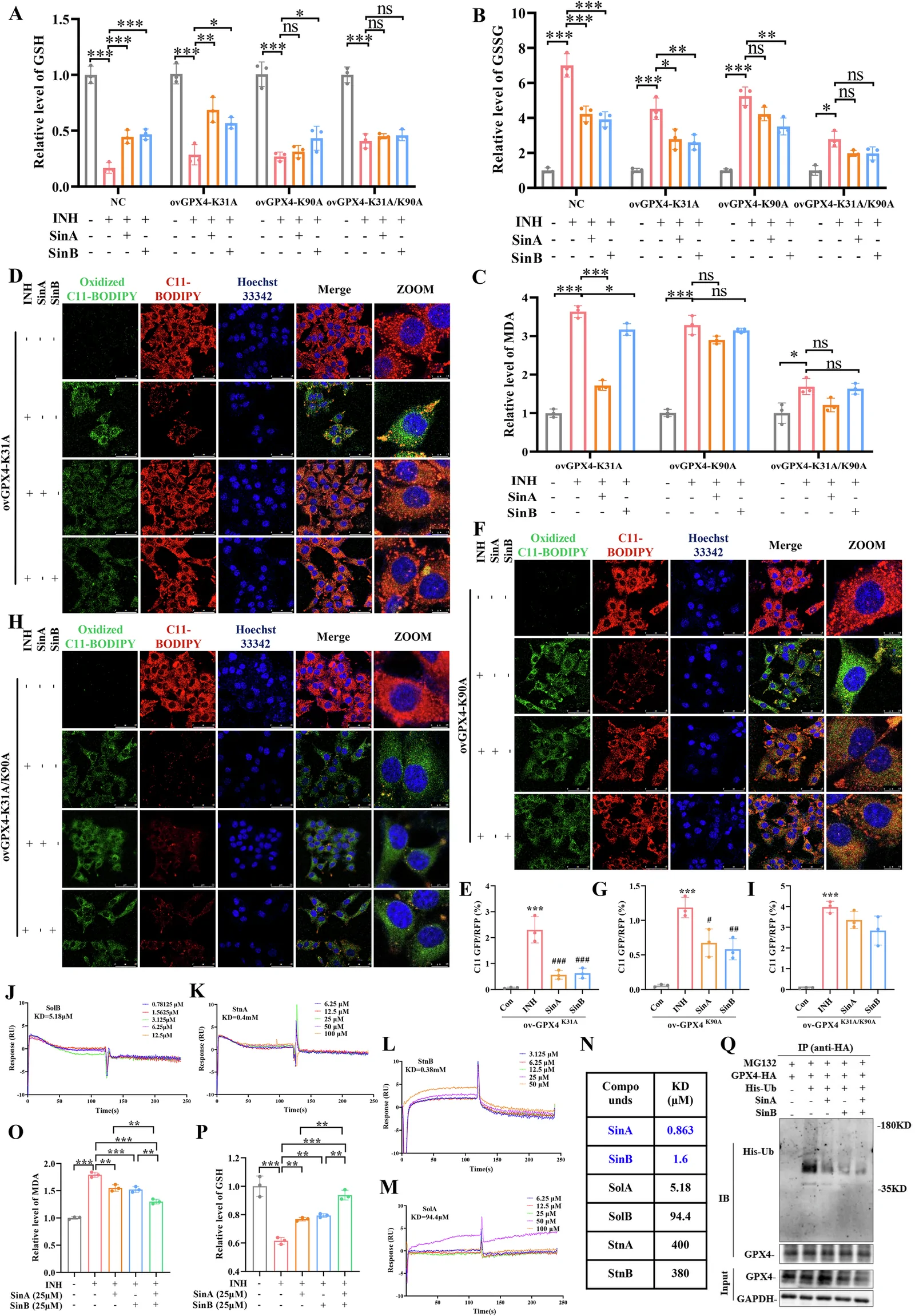
Mutations at K31 and K90 attenuate the functional efficacy of SinA/SinB. **A** GSH levels in WRL68 cell (*n* = 3). **B** GSSG levels in WRL68 cell (*n* = 3). **C** MDA levels in WRL68 cells (*n* = 3). **D**, **E** Laser scanning confocal microscopy detection of C11-BODIPY and oxidized C11-BODIPY fluorescence levels in WRL68 Cells after GPX4-K31A overexpression (scale bar: 50 μm, *n* = 3). **F**, **G** Laser scanning confocal microscopy detection of C11-BODIPY and oxidized C11-BODIPY fluorescence levels in WRL68 cells after GPX4-K90A overexpression (scale bar: 50 μm, *n* = 3). **H**, **I** Laser scanning confocal microscopy detection of C11-BODIPY and oxidized C11-BODIPY fluorescence levels in WRL68 Cells after GPX-K31A/K90A overexpression (scale bar: 50 μm, *n* = 3). **J**–**M** The affinities of GPX4 binding with SolA, SolB, StnA, and StnB were detected by Biacore. **N** The affinities of GPX4 binding with SinA, SinB, SolA, SolB, StnA, and StnB. **O** MDA levels in WRL68 cell (*n* = 3). **P** GSH levels in WRL68 cells (*n* = 3). **Q** Western blot analysis of GPX4 ubiquitination levels in WRL68 cells (*n* = 3). Data were mean ± SD. ^*^P < 0.05, ^**^P < 0.01, ^***^P < 0.001 vs. corresponding groups. NS not significant.

Functional assays in cells expressing GPX4 mutants confirmed the significance of these residues. INH-induced oxidative damage (increased GSSG, decreased GSH, elevated MDA) was effectively counteracted by SinA/SinB in cells expressing GPX4 WT (Fig. 8A–C). This protective effect was weakened in GPX4-K31A cells for SinB, and abolished in GPX4-K90A cells for both compounds. In GPX4-K31A/K90A double mutant cells, the protective effects of both lignans were completely lost (Fig. 8A–C). Similarly, the ability of SinA/SinB to reduce INH-induced lipid peroxidation (C11-BODIPY assay) was diminished or abolished in the mutant cell lines (Fig. 8D–I). To further demonstrate binding specificity, we generated a GPX4-K125A mutant (selecting a distal lysine residue, Lys125 (Fig. S3A), predicted not to participate in binding) and assessed the functional effects of SinA/SinB on this mutant (GSH, MDA, C11-BODIPY). Results showed that INH-induced oxidative damage (increased GSSG, decreased GSH, elevated MDA) was effectively counteracted by SinA/SinB in cells expressing GPX4-K125A (Fig. S3B–D). Similarly, SinA/SinB reduce INH-induced lipid peroxidation (C11-BODIPY assay) in cells expressing GPX4-K125A (Fig. S3E, F). SPR analysis confirmed that other Schisandra lignans (SolA, SolB, StnA, and StnB) had markedly weaker affinities for GPX4 (KD from 5.18 to 0.4 mM) compared to SinA/SinB (Fig. 8J–N), correlating with their lesser protective potency observed initially. Finally, we evaluated the potential synergistic effect of SinA/SinB co-administration. Treating INH-injured WRL68 cells with a combination of 25 μM SinA and 25 μM SinB resulted in a more effective reduction of MDA levels (Fig. 8O) and restoration of GSH levels (Fig. 8P) compared to either compound alone. Furthermore, the combination treatment more potently alleviated INH-induced GPX4 ubiquitination (Fig. 8Q). These data support that SinA/SinB act through a common GPX4-stabilizing mechanism and that their combined use may offer enhanced hepatoprotection against INH-induced injury.

### TRIM25 mediates GPX4 degradation for ferroptosis

**Fig. 9:**
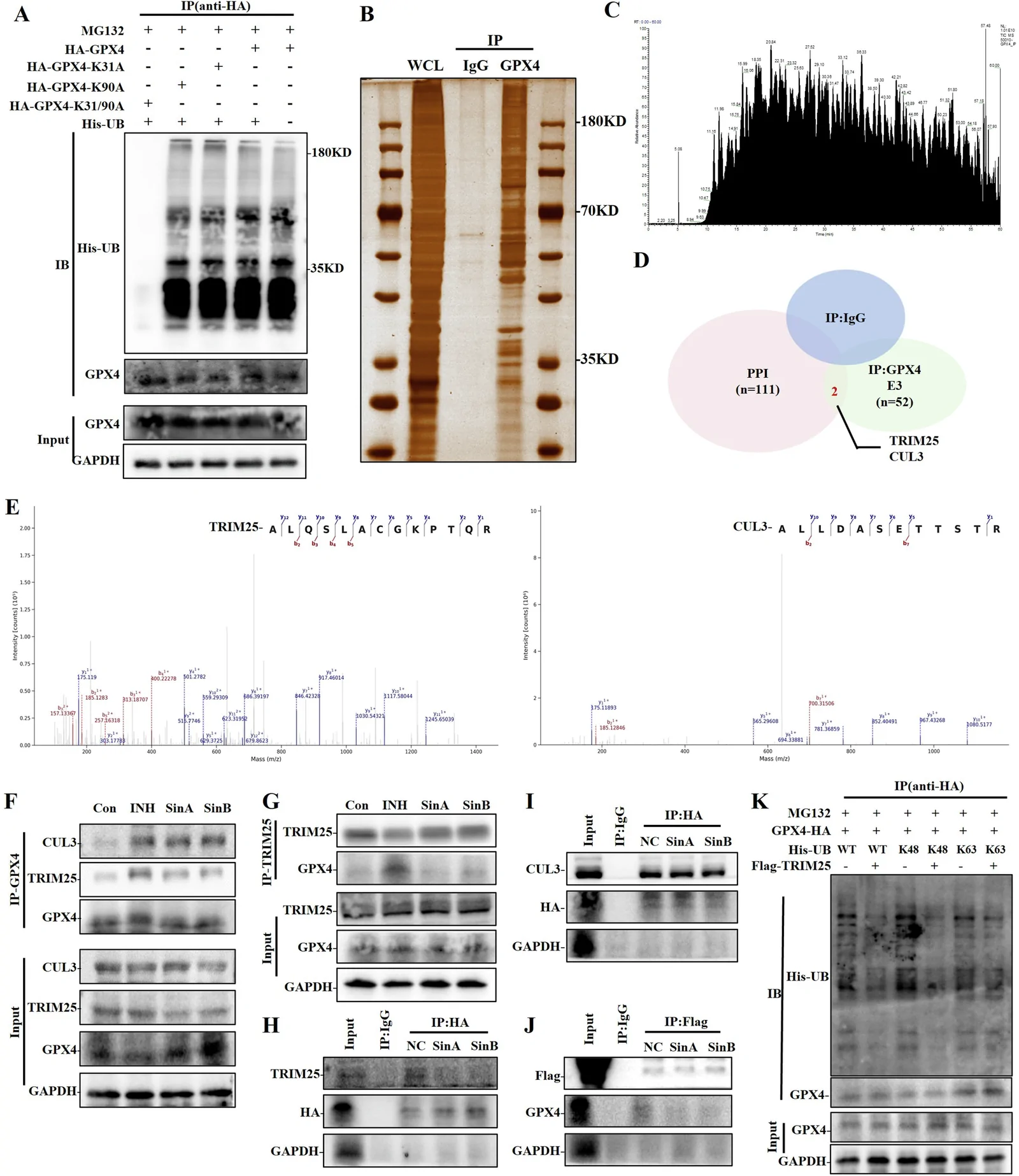
TRIM25 mediates GPX4 degradation for ferroptosis. **A** Co-IP analysis of the ubiquitination of GPX4 WT and and its mutants (K31A, K90A, and K31A/K90A) (*n* = 3). **B** Silver staining of Co-IP assay using anti-gpx4 in WRL68 cells. **C** The pulled-down proteins were identified by mass spectrometry analysis. **D** Venn diagrams of the number of GPX4-binding proteins in the public protein–protein interaction database and E3 identified in WRL68 cells from mass spectrometry assay. **E** Mass spectrometry identification of CUL3 and TRIM25 protein from Co-IP. **F**, **G** Co-IP analysis of the interaction between CUL3/TRIM25 and GPX4 in liver tissue (*n* = 3). **H**–**J** Co-IP analysis of the interaction between Flag-CUL3/ Flag-TRIM25 and HA-GPX4 in HEK293T cells (*n* = 3). **K** HEK293T cells were transfected with HA-GPX4 and His-Ub (WT, K48, or K63) with or without Flag-TRIM25 (*n* = 3).

It has been shown that SinA/SinB-induced early ubiquitination of GPX4 leads to proteasomal (not autophagic) degradation. To test whether K31 and K90 are ubiquitination sites, HA-GPX4 (WT, K31, K90, or K31/K90) was co-transfected with His-ubiquitin (His-Ub) into HEK293T cells. Mutations at K31 or K90 did not affect GPX4 ubiquitination (Fig. 9A). Because E3 ubiquitin ligases catalyze the transfer of ubiquitin on substrate proteins, we used immunoprecipitation in combination with mass spectrometry (IP-MS) to identify the key E3 ligases responsible for proteasomal degradation of GPX4. For this purpose, the Co-IP experiments were conducted and a distinct band was detected (Fig. 9B). Mass spectrometry was used for determining the proteins that might bind to GPX4 (Fig. 9C). IP-MS identified GPX4-binding proteins in WRL68 cells, including 52 E3 enzymes. Among them, cullin-ring ligase 3 (CUL3) and TRIM25 were also listed as GPX4-binding proteins in a publicly accessible protein–protein interaction database (http://iid.ophid.utoronto.ca/ #analyze_PPIs) (Fig. 9D, E). Co-IP assays further verified that CUL3 and TRIM25 directly binds to GPX4 in liver tissue. However, only TRIM25 showed a weakened binding to GPX4 after administration of SinA/SinB (Fig. 9F, G). Similarly, Co-IP assays confirmed a direct interaction between TRIM25 and GPX4, and further revealed that SinA/SinB treatment disrupts this protein complex in WRL68 cells (Fig. 9H–J). TRIM25 can catalyze the formation of K48- or K63-linked ubiquitin chains [42, 43]. To clarify the type of polyubiquitination, HA-GPX4, together with His-Ub (WT, K48, or K63), were transfected into HEK293T cells. TRIM25 mainly induced immunoprecipitation-detectable K48 linked, and not K63-linked, polyubiquitination of GPX4 (Fig. 9K). Collectively, these results demonstrate that the physical interaction between TRIM25 and GPX4 is specifically disrupted upon SinA/SinB treatment. Further ubiquitin chain typing assays verify that TRIM25 predominantly catalyzes K48-linked polyubiquitination of GPX4, thereby governing the proteasomal degradation of GPX4.

### SinB effectively reduces the stability of the GPX4-TRIM25 complex

**Fig. 10:**
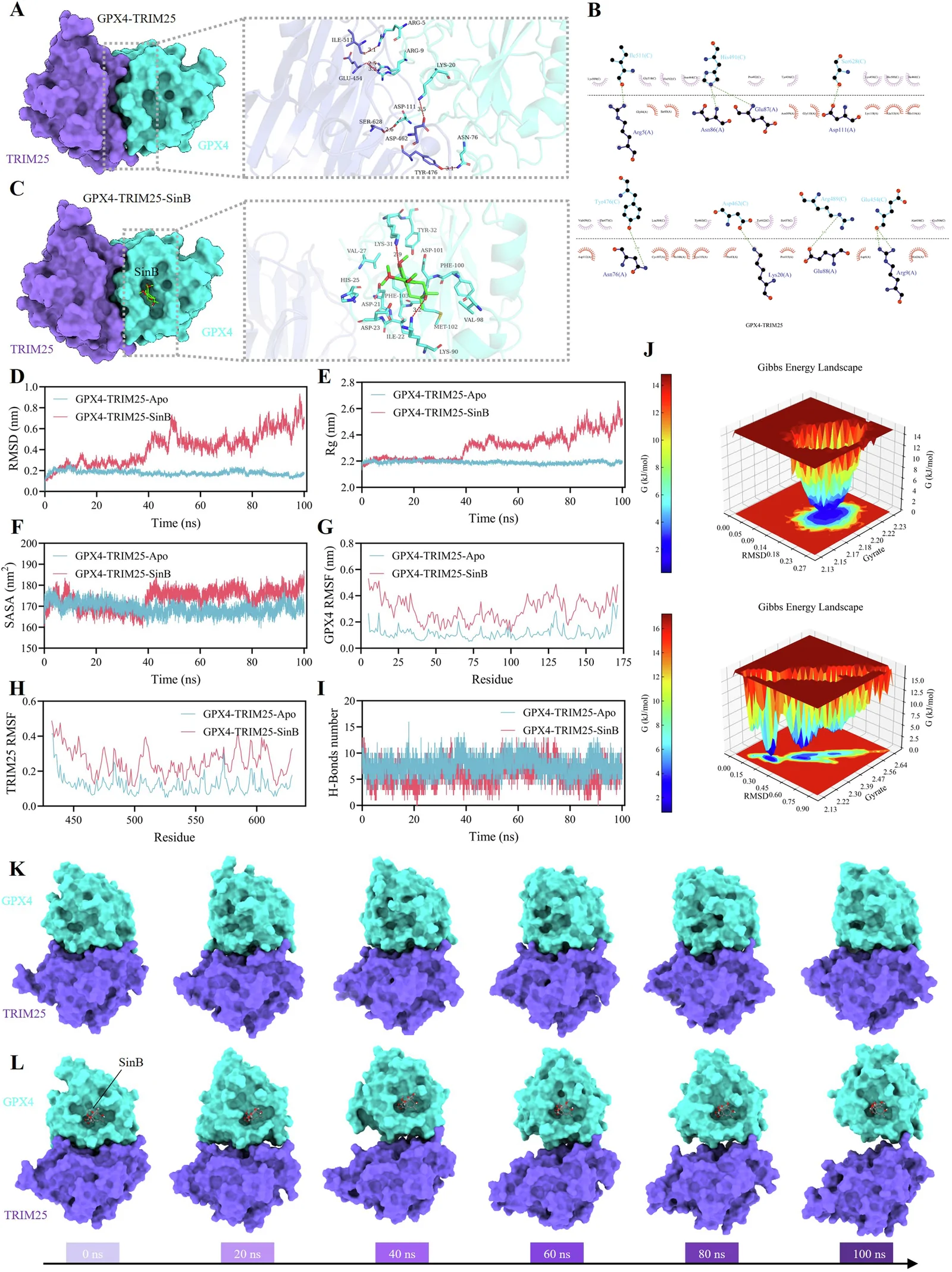
SinB reduces the stability of the GPX4-TRIM25 complex. **A** Model of the GPX4-TRIM25 complex obtained by GRAMM protein–protein docking. GPX4 is shown in blue, and TRIM25 in purple. **B** Two-dimensional visualization of the interfacial interactions between GPX4 and TRIM25. Chain A represents GPX4, and chain C represents TRIM25. Hydrogen bonds are indicated by green dashed lines (GPX4/TRIM25). **C** Overall structure of the GPX4-TRIM25-SinB ternary complex, showing SinB located in the surface pocket of GPX4 and close to the GPX4-TRIM25 interaction interface. SinB is displayed as gray sticks. **D** RMSD changes of the system during 100 ns molecular dynamics simulation. **E** Rg changes of the system during 100 ns molecular dynamics simulation. **F** SASA changes of the system during 100 ns molecular dynamics simulation. **G**, **H** RMSF of GPX4 and TRIM25 during 100 ns molecular dynamics simulation. **I** Number of hydrogen bonds between GPX4 and TRIM25 over time during 100 ns molecular dynamics simulation. **J** Three-dimensional Gibbs free energy landscape. **K**, **L** Representative conformational snapshots taken during the molecular dynamics simulation.

To further elucidate how SinB regulates GPX4-TRIM25 complex stability, this study combined protein–protein docking, binding pattern analysis, and 100 ns MD simulations. Docking results showed that in the apo state, GPX4 and TRIM25 form a compact interface with multiple hydrogen bonds and polar interactions favoring stability (Fig. 10A, B). Upon SinB binding, SinB embedded into GPX4 near the TRIM25 interface, perturbing local recognition space (Fig. 10C). Apo system root mean square deviation (RMSD) remained low, indicating stable conformation. In contrast, RMSD of the GPX4-TRIM25-SinB system increased markedly after ~40 ns and fluctuated up to 0.8–0.9 nm, suggesting SinB-induced rearrangement and reduced stability (Fig. 10D). Apo radius of gyration (Rg was stable at ~2.2 nm, indicating compactness. SinB system Rg increased to 2.4–2.6 nm, indicating expansion and loosening of the interface (Fig. 10E). SinB system solvent accessible surface area (SASA) was higher than apo, especially in the latter half of the simulation, implying reduced protein–protein contact (Fig. 10F). Root mean square fluctuation (RMSF) of both GPX4 and TRIM25 were higher in the SinB system, with pronounced fluctuations at interface-related residues, indicating that SinB affects local GPX4 conformation and propagates this effect to the interface (Fig. 10G, H). Apo system maintained many hydrogen bonds between GPX4 and TRIM25, indicating stable interaction. In the SinB system, hydrogen bond numbers decreased overall (Fig. 10I), suggesting weakened polar networks. The free energy landscape showed a concentrated deep valley for apo, while the SinB system minimum shifted toward higher RMSD and Rg, indicating a looser, less stable thermodynamic state (Fig. 10J). Simulation snapshots (Fig. 10K) showed that in apo, GPX4 and TRIM25 maintained stable binding with continuous interface contact. In the SinB system, their relative orientation shifted, the interfacial gap widened, and SinB remained persistently near GPX4, acting as a conformational perturbing factor (Fig. 10L). Taken together, SinB binds GPX4 near the TRIM25 recognition interface, weakens hydrogen bonds and interfacial contacts, increases RMSD, Rg, SASA, and RMSF, and shifts the lowest-free-energy conformation to a looser, more unstable state, indicating that SinB effectively reduces GPX4-TRIM25 complex stability likely through competitive occupancy and allosteric perturbation.

## Discussion

*Schisandra chinensis*, a traditional medicinal herb with a long history, has garnered significant research attention for its therapeutic potential in chronic liver diseases [22]. Its core active components, lignans, possess multiple pharmacological properties, including antioxidant activity, calcium channel modulation, antitumor effects, hepatoprotection, and cardiovascular support. Studies have shown that Schisandra lignans can mitigate acetaminophen-induced hepatotoxicity by inhibiting GSH depletion and cytochrome P450-mediated metabolic activation [9], and can alleviate inflammation, apoptosis, and oxidative stress by modulating the MAPK pathway, demonstrating potential against hepatic ischemia-reperfusion injury in preclinical models [18]. Among these lignans, SinA and SinB are key bioactive constituents bridging traditional applications and modern pharmacological exploration.

Ferroptosis is a recently recognized form of regulated cell death triggered by iron overload and the accumulation of lipid peroxidation products. Our work provides evidence that INH can initiate ferroptosis in hepatocytes. This process is characterized by excessive lipid peroxidation, elevated malondialdehyde (MDA) levels, and mitochondrial ultrastructural abnormalities—including swelling, vacuolization, and cristae disruption—leading to mitochondrial dysfunction marked by reduced membrane potential and impaired respiratory capacity [21, 23]. More importantly, we further elucidate that INH significantly suppresses GPX4 expression both in vivo and in vitro, thereby inducing ferroptosis, offering novel insights into the molecular mechanisms underlying INH-induced hepatotoxicity [22]. Building on this, we found that SinA/SinB significantly attenuate the extent of INH-induced ferroptosis and improve associated liver injury phenotypes, supporting their potential as anti-ferroptotic agents.

GPX4 is a central regulator of ferroptosis, maintaining the redox balance of cell membranes by reducing lipid hydroperoxides. Recent studies indicate that developing GPX4 activators to inhibit ferroptosis remains challenging. Although potential GPX4 activators (e.g., compound C3) have been proposed, their selectivity, druggability, and clinical applicability remain limited [44]. To establish the essential role of GPX4 in the actions of SinA/SinB, we performed gain- and loss-of-function experiments. When GPX4 was knocked down using siRNA, the protective effects of SinA/SinB against INH-induced ferroptosis markers (MDA, lipid ROS, mitochondrial membrane potential) were completely abolished. Conversely, GPX4 overexpression enhanced the anti-ferroptotic efficacy of SinA/SinB.

To further elucidate the molecular basis of the interaction, we employed molecular docking, SPR, and CETSA to confirm the direct binding of SinA/SinB to the GPX4 protein. Conservation analysis and site-directed mutagenesis revealed that the highly conserved K31 and K90 residues on GPX4 are critical binding sites. SinA primarily relies on K90, while SinB interacts with both K31 and K90. Mutating these lysines to alanine significantly weakened or completely abolished the stabilizing effect of SinA/SinB on GPX4 and their subsequent anti-ferroptotic efficacy, structurally defining the interaction sites. Unlike transcriptional activators (e.g., Nrf2 inducers) or indirect inhibitors of GPX4 degradation, SinA/SinB represent a first-in-class class of site-specific GPX4 stabilizers with defined binding determinants. These results confirm that GPX4 is an indispensable molecular target for the hepatoprotective effects of SinA/SinB. Moreover, this study demonstrates that SinA/SinB do not act through conventional transcriptional or translational activation, but rather directly bind to and stabilize the GPX4 protein. Due to the relatively low content of GPX4 in liver tissue (compared to the in vitro overexpression system) and the rapid in vivo degradation of ubiquitinated proteins in the proteasome, the sampling window is very narrow, and no in vivo ubiquitination CO-IP results were obtained. However, the in vitro data suggest that SinA/SinB binds to GPX4, reducing its interaction with the E3 ubiquitin ligase TRIM25, thereby inhibiting the ubiquitination of GPX4 and ultimately increasing the intracellular level of GPX4. Although K31 and K90 are not themselves ubiquitination sites on GPX4, the binding of SinA/SinB to these residues induces conformational changes in GPX4 that weaken TRIM25 binding. This unique “stabilizer” mechanism provides a new strategy for the development of ferroptosis inhibitors targeting GPX4. Notably, the combined use of SinA/SinB exhibited synergistic effects superior to either compound alone in reducing MDA, restoring GSH, and attenuating GPX4 ubiquitination, suggesting that multi-component extracts of *Schisandra chinensis* may possess greater therapeutic potential.

Although this study reveals the promising therapeutic potential of SinA/SinB against INH-induced liver injury, clinical translation faces challenges. Schisandra lignans generally exhibit low oral bioavailability due to poor solubility, significant first-pass metabolism, and potential efflux by transporters such as P-glycoprotein [45, 46]. Nevertheless, the doses used in our study are within the range of reported pharmacologically effective doses in rodent models of liver injury [47, 48]. Furthermore, the INH monotherapy-induced liver injury model used here, while valuable, does not replicate the complex environment of multi-drug regimens used in clinical tuberculosis treatment. Future research should develop models and explore novel drug delivery systems to improve the pharmacokinetic profiles of lignans and facilitate their translation into clinical applications.

## Conclusion

In summary, this study demonstrates that SinA/SinB directly bind to the K31 and K90 residues of GPX4, reduce the binding of the E3 ligase TRIM25, inhibit the ubiquitin‑dependent degradation of GPX4, suppress ferroptosis, and ameliorate INH‑induced liver injury. These findings not only reveal a novel mechanism underlying INH hepatotoxicity but also provide new lead compounds and a theoretical foundation for the development of anti‑ferroptotic drugs targeting GPX4.

## Materials and methods

### Chemicals and reagents

INH (CAS 54-85-3) was procured from Sigma-Aldrich (St Louis, MO, USA). SinA (CAS 61281-38-7), Schisandrin B (SinB, CAS 61281-37-6), Schisandrol A (SolA, CAS 7432-28-2), Schisandrol B (SolB, CAS 58546-54-6), Schisantherin A (StnA, CAS 58546-56-8), and Schisantherin B (StnB, CAS 58546-55-7) were all acquired from Chengdu Manster Biotechnology Co., Ltd (Chengdu, China). The purities of these compounds were determined to be >98% by the HPLC method. Bifendate (DDB, CAS 73536-69-3) was obtained from Sichuan Weikeqi Pharmaceutical Co., Ltd (Sichuan, China). High-glucose Dulbecco’s Modified Eagle Medium (DMEM) and penicillin-streptomycin were procured from Gibco (Carlsbad, CA, USA). Trypsin and fetal bovine serum (FBS) were acquired from ExCell Bio (Jiangsu, China). Phosphate-buffered saline (PBS) was obtained from Nanjing BioChannel Biotechnology Co., Ltd (Nanjing, China). 3-(4,5-dimethylthiazol 2-yl)-2,5-diphenyltetrazolium bromide (MTT) was purchased from Shanghai Acmec Biochemical Technology Co., Ltd (Shanghai, China). Kits for aspartate aminotransferase (AST), alanine aminotransferase (ALT), and GSH were bought from Nanjing Jiancheng Bioengineering Institute (Nanjing, China). The malondialdehyde (MDA) kit and Hoechst 33342 were acquired from Beyotime Biotechnology (Shanghai, China). The FeRhoNox-1 fluorescent probe was obtained from Glpbio (Monclair, CA, USA). BODIPY™ 581/591 C11 was bought from Thermo Fisher Scientific (Waltham, MA, USA). JC-1 dye was acquired from Yeasen (Shanghai, China). DAPI stain was bought from Servicebio (Wuhan, China). Antibodies to GPX4 (T56959S) and SCL7A11 (T57046XF) were obtained from Abmart Shanghai Co., Ltd (Shanghai, China). CUL3 (YT1159) and tripartite motif containing 25 (TRIM25) (YM960144) were purchased from Immunoway (Jiangsu, China). GAPDH (10494-1-AP), Keap1 (10503-2-AP), Nrf2 (16396-1-AP) and β-actin (66009-1-Ig) were obtained from Proteintech (Wuhan, China). Ferrostatin-1 (Fer-1) (HY-100579), Erastin (HY-115594) and RSL-3 (HY-100218A) were purchased from MedChemExpress (Monmouth Junction, USA).

### Cell culture

WRL68 cells were cultivated in DMEM high-glucose medium that also contained 10% FBS and 1% penicillin-streptomycin solution. Both cell lines were maintained in an incubator with 5% CO_2_ at 37 °C under saturated humidity. Subculturing was initiated when cell confluence exceeded 80%, followed by medium replacement 1 day after subculturing.

### Animals and experimental design

The animal experiments in this study were conducted in accordance with relevant animal care laws and guidelines. All procedures were approved by the Guangzhou University of Chinese Medicine Laboratory Animal Ethics Committee (N0.20230811). C57BL/6 mice, aged 4 to 6 weeks, were obtained from the Guangdong Animal Testing Center. The mice were housed under standard conditions, with a temperature of 25 ± 2 °C and a relative humidity of 50 ± 5%. A 12-h on/off lighting cycle was implemented using incandescent lamps. The mice were provided with ad libitum access to feed and water throughout the study.

SPF-grade male C57BL/6 mice were randomly divided into nine groups, including a normal control group, an INH group, a SinA (Low) group, a SinA (Middle) group, a SinA (High) group, a SinB (Low) group, a SinB (Middle) group and a SinB (High) group with six mice in each group. Except for the normal control group, which received intragastric administrations of saline, all other groups received 150 mg/kg of INH daily to establish a mouse model of liver injury. Two hours after each INH administration, the normal control group and INH group received saline intragastrically, and the SinA/SinB groups received 25, 50, and 100 mg/kg of the respective drugs. All mice were gavaged with an equal volume of 0.1 ml/10 g of the respective treatments daily for 20 consecutive days. The maximal tumor burden permitted was not applicable, as this is not a tumor study.

### MTT assay

WRL68 cells were inoculated into 96-well plates at a density of 5 × 10^3^ cells per well. Once the cells adhered to the plate, they were treated with INH at concentrations of 12.5, 25, 50, and 100 mM, with 100 μL/well and six replicate wells for each concentration. Cell viability was determined by adding MTT (0.5 mg/mL) to each well and incubating for 4 h at 37 °C. Subsequently, formazan crystal products were dissolved in dimethylsulfoxide (DMSO) for 10 min and the absorbance value was measured at 570 nm with a microplate reader (Bio-Rad Laboratories, Hercules, CA, USA).

### ALT or AST assay

Hepatic function indicators in the cells were determined using ALT and AST assay kits. According to the instructions, cellular samples were collected and incubated with the respective ALT and AST working solutions at 37 °C. The absorbance values were then measured at 510 nm using a microplate reader.

### Protoporphyrin IX (PPIX) content determination

Owing to the absence of endogenous precursors for PPIX, such as 5-aminolevulinic acid (ALA) (A0325, TCI, Shanghai, China), in cells, exogenous 5-ALA was added to stimulate the production of protoporphyrin. WRL68 cells were inoculated into confocal dishes at a density of 1.5 × 10^4^ cells per dish. Once the cells adhered to the dish, modeling and drug administration were carried out. After the incubation, the cells were incubated with 1 mM 5-ALA in the dark for 4 h. Imaging of the cells was subsequently conducted using a laser scanning confocal microscope (Leica TCS SP8 X, Leica, Wetzlar, Germany).

### Fe^2+^ content detection

The intracellular Fe²⁺ content was determined using the FeRhoNox-1 (Fe^2+^ indicator) fluorescent probe. WRL68 cells were inoculated into confocal dishes at a density of 1.5 × 10^4^ cells per dish. The cells were washed with PBS after the incubation and then incubated with 5 μM FeRhoNox-1 working solution in the dark for 1 h. The cells were washed three times with PBS and subsequently incubated with Hoechst 33342 for 10 min. After another three washes, serum-free medium was added to the cells. Images were obtained using a laser scanning confocal microscope with an excitation wavelength of 532 or 543 nm and an emission wavelength of approximately 570 nm.

### Mitochondrial membrane potential analysis

Mitochondrial membrane potential was evaluated using the JC-1 staining method. Cells were inoculated with 50 μg/mL of JC-1 staining working solution for 30 min at 37 °C in the dark. JC-1 monomer fluorescence (green) was detected at an excitation wavelength of 514 nm, while JC-1 aggregate fluorescence (red) was detected at an emission wavelength of 590 nm. Images were obtained using a laser scanning confocal microscope.

### Immunofluorescence analysis

The cells were fixed in 4% paraformaldehyde for 20 min and then permeabilized with 0.5% Triton X-100 for 30 min. After being washed with PBS, the cells were blocked with 5% BSA for 30 min at room temperature, washed three more times with PBS, and probed with the GPX4 antibody overnight at 4 °C. The cells were incubated with the secondary antibody conjugated to Dylight-594 for 2 h at room temperature. After washing three times with PBS, the cells were then incubated with 20 mg/mL DAPI for 20 min. Images were captured (594 nm/618 nm for Dylight-594; 488/519 nm for green fluorescent protein and 340/488 nm for DAPI) using confocal microscopy.

### H&E staining

The liver tissue was fixed in 4% paraformaldehyde for no less than 24 h, followed by dehydration through an ethanol gradient and clarification with xylene before immersing the tissue in wax. Subsequently, the tissue was embedded in paraffin and 4-μm thin sections were made. The sections were placed on slides and baked in a slide warmer for 2 h, then dewaxed with xylene and rehydrated through an ethanol gradient, followed by immersion in distilled water. The sections were stained with hematoxylin for 2 min, differentiated with 1% hydrochloric acid in alcohol, and counterstained with 0.5% eosin for 10 s. They were then dehydrated through an ethanol gradient, immersed in xylene, air-dried, and mounted with neutral resin. Finally, images of the sections were captured under optical microscopes at 10 x and 40 x magnifications.

### Intracellular reactive oxygen species (ROS) measurement

ROS levels in WRL68 cells were evaluated using the ROS Detection Kit (MA0219, Dalian, China) in accordance with the provided guidelines. Briefly, cells were stained with 10 μM 2′, 7′-dichlorodihydrofluorescein diacetate (DCFH-DA) at 37 °C for 20 min in the dark, and washed three times with PBS. After staining, the levels of ROS production were detected by fluorescence microscopy (Leica TCS SP8, Germany).

### Western blotting

Cells were homogenized with the ice-cold RIPA buffer for 30 min to provide whole cell lysates. Concentrations of total proteins were determined using an enhanced BCA protein assay kit (DQ111-01, TransGen Biotech, Beijing, China). Lysates were separated by 8–12% sodium dodecyl sulfate polyacrylamide gel (SDS-PAGE) and subsequently transferred to PVDF membranes. Membranes were blocked in 5% non-fat milk, probed with primary antibody (1:1000 diluted) for 2 h, and further incubated with the secondary antibody (1:3000 diluted) for 1 h. Then, membranes were washed and developed with an enhanced chemiluminescence substrate. Images were captured using a Tanon 5200 Imaging Analysis System (Tanon, Shanghai, China). Relative protein expressions were shown by densitometry analysis using the ImageJ software.

### RT-qPCR assay

The total RNA from the cells or liver tissues was extracted using the Trizol method. Subsequently, cDNA was generated from the total RNA utilizing a reverse transcription kit (RR037A, TaKaRa, Japan) in accordance with the provided instructions. Real-time PCR was conducted as outlined employing a SYBR® Green Pro Taq HS qPCR Kit (RR430S, Accurate Biology, Hunan, China). The (human) *Gpx4* primers: forward primer 5’-GTGGATGAAGATCCAACCC-3’ and reverse primer 5’-TTGTCGATGAGGAACTTGG-3’; (human) *Gadph* primers: forward primer 5’-GAGTCAACGGATTTGGTCGT-3’ and reverse primer 5’- GACAAGCTTCCCGTTCTCAG-3’; (mouse) *Gpx4* primers: forward primer 5’-AAAGTCCTAGGAAACGCCCG-3’ and reverse primer 5’-CCCTTGGGCTGGACTTTCAT-3’; (mouse) *Keap1* primers: forward primer 5’-AAGTGTGAGATCCTGCAGGC-3’ and reverse primer 5’-CGACTAGATGCCACTCGTCC-3’; (mouse) *Nrf2* primers: forward primer 5’-ACAGTCCCAGCAGAGTGATG-3’ and reverse primer 5’-CACACACTTTCTGCGTGCTC-3’, (mouse) *SCL7A11* primers: forward primer 5’-GAGTCTGGGTGGAACTGCTC-3’ and reverse primer 5’-AGAGGGCAACAAAGATCGGG-3’; (mouse) *Gadph* primers: forward primer 5’-GTGTCCGTCGTGGATCTGA-3’ and reverse primer 5’- CCTGCTTCACCACCTTCTTG-3’; (mouse) *Fsp1* primers: forward primer 5’-ACCGCAGTGCATTTGAGAGT-3’ and reverse primer 5’- GGCCTCTGCTTCATGGAGTT-3’; (mouse) *Acsl4* primers: forward primer 5’-ACTGGCGATATTGGAGAAT-3’ and reverse primer 5’- CACATAGGACTGGTCACTT-3’.

### siRNA interference and plasmid transfection

The siRNA sequences targeting GPX4 (siGPX4) and the control siRNA (siCon) were custom-synthesized by Suzhou Genepharma Co., Ltd (Suzhou, China). The sense sequences for GPX4 siRNA-1 (siGPX4-1), siRNA-2 (siGPX4-2) and siRNA-3 (siGPX4-3) are 5’-CAGGGAGUAACGAAGAGAU-3’, 5’-GTGGATGAAGATCCAACCCAA-3’ and 5’-GCACATGGTTAACCTGGACAA-3’, respectively. The overexpressing plasmid of GPX4 (pcDNA3.1-GPX4) and the control vector (pcDNA3.1) were obtained from Obio Technology (Shanghai) Corp., Ltd. All siRNAs and plasmids were transfected into the cells using Lipofectamine™ 3000 Transfection Reagent following the manufacturer’s instructions for a duration of 48 h.

### Co-immunoprecipitation (Co-IP) analysis

Cells were lysed using pre-chilled NP40 (BL653A, Biosharp, Anhui, China), and the supernatant obtained after centrifugation was quantified. A portion of this supernatant was used as Input for subsequent Western blot experiments. The remaining total cellular protein, quantified to 250 μg, was mixed with either the GPX4 antibody (1:200) or normal IgG (1:200) and incubated on a rocker at 4 °C for 2 h. Following incubation, 40 μL of agarose beads were added and incubated overnight on a rocker at 4 °C. After overnight incubation, the mixture was centrifuged, and the beads were washed three times with PBS. Subsequently, 70 μL of 1×SDS-PAGE Sample Loading Buffer was added, and the samples were heated at 100 °C for 5 min. Ubiquitination was then detected by Western blot.

### Molecular docking analysis

Molecular docking was conducted with AutoDock Vina 1.1.2, preparing the receptor protein with PyMol 2.52 by removing water molecules, ions, and small molecules, and setting up a docking box to encompass the entire protein. The highest scoring docked conformation was identified as the binding conformation and visualized using PyMol 2.5.2. The GPX4 (PDB: 6HN3). The 3D structure of SinA/SinB were downloaded from Pubchem, and their energies were minimized under the MMFF94 force field. The compound CIDs are as follows: SinA (155256) and SinB (108130).

### Surface plasmon resonance (SPR) assay

The interaction between GPX4 and the compounds SinA/SinB was assessed using the BiacoreX100 system (GE Healthcare, Uppsala, Sweden) at 25 °C. Recombinant human GPX4 protein was immobilized on a CM5 sensor chip using an amine coupling kit (GE Healthcare, Buckinghamshire, UK), achieving immobilization levels of approximately 8876 RU (Response Units). The compounds were then injected as analytes at various concentrations into a PBS buffer (0.5% DMSO), serving as the running buffer. Binding affinity studies involved injecting analytes at specified concentrations with a flow rate of 30 μL/min, allowing 150 s each for contact and dissociation phases. The chip was subsequently washed with the running buffer between analyses.

### Preparation of recombinant proteins

The cDNA encoding the human cytosolic GPX4 (c-GPX4) was amplified by RT-PCR, and the resulting fragment was ligated into the pET28α (+) vector. The expression strategy was designed in such a way that the complete primary structure of GPX4 (except the starting Met) was retained. An additional tail of 11 amino acids, which included a hexa-histidine-tag and was introduced to allow its effective purification. The final N-terminal sequence of the recombinant protein reads M-R-G-S-H-H-H-H-G-S-A-C, in which the last C corresponds to the initial M of the native enzyme. GPX4 was expressed in *E. coli* Codonplus, and the cells were cultured in the Luria Bertani medium at 37 °C to an optical density of 0.6–0.8 at 600 nm, then induced with 1 mM Isopropyl-β-D-thiogalactopyranoside for 8 h at 25 °C. The protein was purified by nickel affinity chromatography and concentrated using an Amicon filter cartridge (Merck Millipore, USA). The protein was further purified by a Sephadex G-200 size-exclusion column (MicroCal, GE Healthcare, USA) and concentrated to about 5 mg/mL using a 21-kDa concentrator. SDS-PAGE was used to determine the purity of GPX4.

### Isothermal titration calorimetry (ITC) assay

The binding interactions of western blotting with both GPX4-WT and the GPX4-K31R, GPX4-K90R mutants were analyzed by an EAQ-ITC instrument (MicroCal, GE Healthcare, USA) at 25 °C. SinA/SinB was prepared in a buffer containing 20 mM Tris-HCl (pH 7.5) and 150 mmol/L NaCl. Then, 0.1 mM SinA/SinB was injected 19 times in 60-μl aliquots from the syringe into a sample cell containing 300 μl of 0.4 mM GPX4. The resulting data were processed using the Origin 7.0 software, fitting to a two-site binding model to determine thermodynamic parameters such as enthalpy change (Δ*H*), entropy change (Δ*S*), and the equilibrium dissociation constant (*KD*).

### Cellular thermal shift assay (CETSA)

Purified recombinant GPX4 (6 mg/mL) was incubated with SinA/SinB (100 μMl/L) for 30 min at 25 °C. The supernatant was evenly divided into six tubes according to 18 μg per tube and heated at 45, 50, 55, 60, 65, and 70 °C, respectively, for 10 min, after which the supernatant was obtained by centrifugation for western blotting analysis.

### Drug affinity responsive target stability (DARTS) assay

Purified recombinant GPX4 (6 mg/mL) was diluted 1:10 with the TNC buffer (50 mM Tris-HCl, pH 8.0, 50 mM NaCl, 10 mM CaCl_2_) and treated with SinA/SinB of various concentrations, or DMSO as a control, for 1 h at room temperature. Pronase (25 μg/mL) was then added and incubated for 30 min at 37 °C. The reactions were stopped by adding the SDS-PAGE loading buffer, and the samples were analyzed via western blotting using an anti-GPX4 antibody.

### GSH detection

The GSH and glutathione, oxidized (GSSG) were measured using the GSH and GSSG assay kits. Following treatment, the cells were rinsed with PBS and then collected by centrifugation, and the supernatant was discarded. Cell lysis was achieved by a protein removal reagent three times the volume of the cell pellet, followed by two cycles of freeze-thawing using liquid nitrogen and a water bath at 37 °C. The lysates were then cooled for 5 min at 4 °C and centrifuged at 10,000×*g* for 10 min. The clear supernatant obtained was used to measure the total GSH levels. To quantify GSSG, the supernatant samples were treated with a diluted GSH clearing aid (20 μL/100 μL of the sample) and GSH clearing working solution (4 μL/100 μL of the sample), followed by an incubation at 25 °C for 60 min. The absorbance was measured at 412 nm after an additional 25 min. The concentration of GSH in the samples was determined by comparing the absorbance values to a standard curve provided with the kit. The concentration of reduced GSH was calculated by subtracting twice the amount of GSSG from the total glutathione measured: GSH = Total glutathione – 2 × GSSG.

### Measurement of MDA content

To determine the level of malondialdehyde, a marker of lipid peroxidation, the MDA assay kit was utilized. Following the drug treatment, the cells were lysed using 100 μL of the protein lysis buffer. According to the instructions provided with the kit, the MDA detection solution was prepared and combined with the cell lysate. This mixture was then heated to 100 °C for 15 min, allowed to cool, and centrifuged at 10,000×*g* for 10 min. A volume of 200 μL of the supernatant was pipetted into a 96-well plate, and the absorbance was measured at 532 nm. The concentration of MDA was calculated based on a standard curve and normalized to the protein concentration in the samples.

### Intracellular BODIPY 581/591 C11 staining

Lipid peroxidation within cells was determined using the BODIPY 581/591 C11 probe. The cells were plated at a density of 2.0 × 10^4^ cells/well in confocal dishes. Following treatment, the cells were incubated with 50 μL of Hank’s Balanced Salt Solution containing 40 nM BODIPY 581/591 C11 for 30 min at 37 °C. Fluorescence microscopy images were obtained using a laser scanning confocal microscope. The ratio of green fluorescent protein to red fluorescence protein, which reflects the oxidation of BODIPY 581/591 C11, was analyzed and quantified using the ImageJ software.

### Cell viability assay

Calcein/PI Assay Kit (C2015M, Shanghai, China) was used to detect cell viability. WRL68 cells were seeded in 96-well plates and stimulated with INH for 24 h, subsequently treated with different Schisandrin lignans (25 μM) for 24 h. Then, Calcein-AM and PI reagents were added and incubated in the dark at 37 °C for 20 min. Cell viability was calculated based on the absorbance reading at 494 and 535 nm.

### Molecular dynamics simulation (MD)

In this study, all-atom molecular dynamics (MD) simulations of 100 ns were performed on the protein-small molecule complexes obtained from molecular docking and on the apo protein using GROMACS [49]. The detailed procedures and parameter settings for the MD simulations were as follows: (1) The pdb2gmx tool in GROMACS was used to convert the PDB file into a coordinate file in gro format and to generate the corresponding topology file (.top) and force field parameter files, with the AMBER99SB-ILDN force field selected for the protein. For the small molecule, pretreatment was carried out using AmberTools22 to assign the GAFF [50], and Gaussian 16 W was employed to add hydrogen atoms and to calculate the RESP (Restrained Electrostatic Potential) charges. The resulting electrostatic potential data were incorporated into the topology file of the MD system. (2) The protein-small molecule complex was solvated using the TIP3P (Three-point transferable intermolecular potential) water model, with a minimum distance of 1.2 nm between the protein atoms and the edge of the water box. An appropriate number of Na⁺ counterions were added to neutralize the charge of the simulation system. (3) To obtain a stable system, energy minimization was performed using the steepest descent algorithm. (4) For short-range electrostatic and van der Waals interactions, a cutoff distance of 1.0 nm was applied. Long-range electrostatic interactions were handled using the Particle Mesh Ewald method. All hydrogen bonds were constrained using the LINCS algorithm. (5) During MD simulations, pressure was maintained at 1 bar using the Berendsen barostat, and temperature was regulated using the V-rescale thermostat method. (6) Subsequently, the protein structure was subjected to a pre-equilibration of 100 ps to optimize the initial conformation of the protein in the solvent. Equilibration was first performed in the canonical ensemble for 100 ps to gradually heat the protein-solvent system to the target temperature. This was followed by equilibration in the isothermal-isobaric ensemble for 100 ps to equilibrate the pressure of the solvent and protein system. (7) After completing energy minimization and system equilibration, a 100 ns molecular dynamics simulation was carried out for each protein-small molecule complex under periodic boundary conditions in the NPT ensemble. The system temperature was maintained at 300 K and the pressure at 1 bar. The integration time step was set to 2 fs, and trajectories (including coordinates, velocities, and energies) were recorded every 10 ps for subsequent analysis.

### Statistical analysis

The data were expressed as mean ± SD. Statistical analysis was performed using Student’s *t*-test and one-way ANOVA test in SPSS 19.0 software. Graphs were created using GraphPad Prism 6.0 software. A significance level of *P* < 0.05 was considered statistically significant.

## Supplementary information

Supplemental Material

## Data Availability

The datasets used or analyzed during the current study are available from the corresponding author on reasonable request.
