# Supplementary material for: Lys31/Lys90-dependent stabilization of GPX4 by Schisandrin lignans suppresses ferroptosis in drug-induced liver injury
Source: Cell Death Discov. 2026 Aug 17;12:456. doi: 10.1038/s41420-026-03288-4 (PMC13754793; doi:10.1038/s41420-026-03288-4)
Supplement: Supplementary file 1 [file 41420_2026_3288_MOESM1_ESM.docx]

**Supplementary**

**Fig. S1 SinA/SinB inhibit ferroptosis in a GPX4-dependent manner in AML12 cells.** **(A)** Measurement of GSH levels in AML12 cells (n=3). (B) Measurement of MDA levels in AML12 cells (n=3). **(C-D)** Detection of C11-BODIPY and oxidized C11-BODIPY fluorescence levels in AML12 cells using laser scanning confocal microscopy (scale bar: 50 μm, n=3). (E) Western blot analysis of GPX4, ACSL4, SCL7A11 and FSP1 protein expression levels in AML12 cells. (F-G) CETSA experiments of SinA/SinB with GPX4 protein. Data are mean ± SD. **P* < 0.05, ***P* < 0.01, ^***^*P* < 0.001 vs. control group, ^##^*P* < 0.01, ^###^*P* < 0.001 vs. INH group.

**Fig. S2 Erastin induces ferroptosis in WRL68 cells.** (A) Stained living and dead cells after incubation with Erastin (n=3). (B) Western blot analysis of GPX4 and SCL7A11 protein expression levels in the Erastin-induced WRL68 cells (n=3). Data are mean ± SD. **P* < 0.05, ***P* < 0.01, ^***^*P* < 0.001 vs. control group, ^##^*P* < 0.01, ^###^*P* < 0.001 vs. Erasin group.

**Fig. S3 SinA/SinB inhibit ferroptosis in GPX4-K125**. (A) Multiple sequence alignment of GPX4 from different species. *H. sapiens*, *M. musculus*, *P. troglodytes, R. norvegicus and S. scrofa represent Homo sapiens*, *Mus musculus*, *Pan troglodytes* and *Rattus norvegicus, Sus scrofa*, respectively. The red background represents extremely conserved residues, and the red font represents relatively conserved residues. (B) GSSG levels in WRL68 cell. (C) GSH levels in WRL68 cell (n=3). (D) MDA levels in WRL68 cells (n=3). (E-F) Laser scanning confocal microscopy detection of C11-BODIPY and oxidized C11-BODIPY fluorescence levels in WRL68 Cells after GPX4-K125A overexpression (n=3). Data are mean ± SD. ^*^*P <* 0.05, ^**^*P <* 0.01, ^***^*P <* 0.001 vs. corresponding groups. ns. not significant.
